# Identification of Boronate-Containing Diarylpyrimidine Derivatives as Novel HIV-1 Non-Nucleoside Reverse Transcriptase Inhibitors

**DOI:** 10.3390/molecules27217538

**Published:** 2022-11-03

**Authors:** Da Feng, Hao Lin, Liyang Jiang, Zhao Wang, Yanying Sun, Zhongxia Zhou, Erik De Clercq, Christophe Pannecouque, Dongwei Kang, Peng Zhan, Xinyong Liu

**Affiliations:** 1 Department of Medicinal Chemistry, Key Laboratory of Chemical Biology (Ministry of Education), School of Pharmaceutical Sciences, Cheeloo College of Medicine, Shandong University, 44 West Culture Road, Jinan 250012, China; 2 Department of Cancer Center, Shandong University, Jinan 250117, China; 3 Rega Institute for Medical Research, Laboratory of Virology and Chemotherapy, KU Leuven, Herestraat 49 Postbus 1043 (09.A097), B-3000 Leuven, Belgium; 4 China-Belgium Collaborative Research Center for Innovative Antiviral Drugs of Shandong Province, 44 West Culture Road, Jinan 250012, China

**Keywords:** HIV-1, NNRTIs, NNIBP, DAPY, boronate, drug design

## Abstract

In this study, privileged boronic acid ester was introduced into the right wing of etravirine (ETR) to obtain a series of novel boronate-containing derivatives. These newly synthesized derivatives were evaluated for their anti-HIV potency in MT-4 cells using the MTT method, and their inhibitory activity to HIV-1 reverse transcriptase (RT) was assayed by the ELISA method. Most of the synthesized compounds displayed promising antiviral activity against the wild-type and a wide range of HIV-1 mutant strains. In particular, **4a** exhibited the most potent activity against the wild-type and a panel of single mutations (L100I, K103N, Y181C, and E138K) with EC_50_ values ranging from 0.005 to 0.648 μM, which were much superior to those of nevirapine (EC_50_ = 0.151 μM). Moreover, **4b** turned out to be an effective inhibitor against the double-mutant strains F227L + V106A and RES056 with EC_50_ values of 3.21 and 2.30 μM, respectively. RT inhibition activity and molecular docking were also investigated.

## 1. Introduction

**Figure 1:**
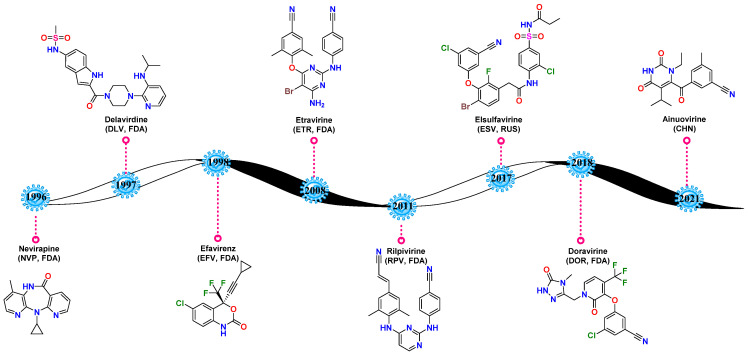
Chemical structures of approved NNRTIs drugs.

Since the first case reported in the United States in 1981 [1], acquired immunodeficiency syndrome (AIDS) has become a major global public health problem which seriously endangers human health. HIV-1, with strong virulence and high mortality, is the main causative agent of AIDS [1]. Reverse transcriptase (RT), which is responsible for reverse transcription of single-stranded RNA into double-stranded DNA, plays an important role in the life cycle of HIV-1. It is unique to the virus and there is no homologous enzyme in human body, making it an ideal target for drug intervention [2]. There are two types of RT inhibitors available on the market: the nucleoside/nucleotide RT inhibitors (NRTIs/NtRTIs) and non-nucleoside RT inhibitors (NNRTIs) [3,4]. Currently, six NNRTIs have been approved by the U.S. Food and Drug Administration (FDA), including the first generation nevirapine (NVP), delavirdine (DLV), and efavirenz (EFV) and the second generation etravirine (ETR), rilpivirine (RPV), and doravirine (DOR) [5]. Moreover, elsulfavirine (ESV) was marketed in Russia for the treatment of HIV in 2017 [6] and ainuovirine (ANV) in China in 2021 [7] (Figure 1). Although NNRTIs are widely used in clinic due to their potent antiviral activity and high selectivity, their effects are compromised by drug resistance (such as Y181C, E138K, and F227L + V106A) and adverse effects (such as hypersensitivity reactions) [8,9,10,11]. Therefore, the development of next-generation NNRTIs with better drug-resistance profiles and lower toxicity is still in high demand.

Recently, a considerable amount of literature has illustrated the diverse applications of boronate-containing motifs in the construction of therapeutically useful bioactive molecules [12]. Up to now, four boronate-containing drugs have been approved for clinical use and more are currently in clinical trials [13]. The increasing interest in boronate-containing compounds is due to their unique binding properties to biological targets; for example, boronic acid is a strong Lewis acid because of the open electronic shell of boron, which means that boronic acids can be converted from a triangular planar sp^2^ boron form to a tetrahedral sp^3^ boron under physiological conditions, thus generating multiple modes when binding to biological targets, such as multiple hydrogen bonding interaction forces and covalent interactions [14]. Therefore, boronic acids and its esters are becoming increasingly prevalent in contemporary drug design. Recent evidence suggests that boronic acid and its esters have been successfully employed in antiviral agents targeting HIV-1 protease and HCV polymerase [15,16,17], which prompted us to shift our antiviral drug research territory to boronate-containing agents [18,19].

**Figure 2:**
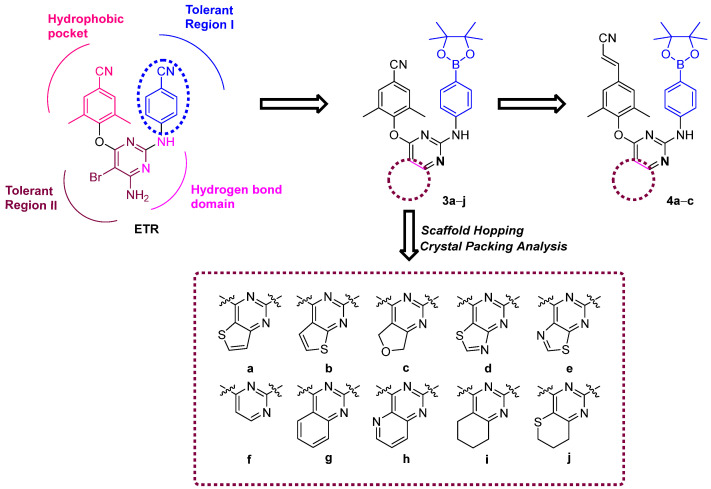
The design strategy of the newly designed phenylboronic acid pinacol ester-containing compounds.

As part of our ongoing research, in this work, the privileged phenylboronic acid pinacol ester was introduced in the right wing of the lead ETR to develop stronger interactions with NNRTIs binding pocket (NNIBP), with the hope of improving the activity and drug-resistance profiles (Figure 2). Meanwhile, according to our previous exploration on the structure–activity relationships (SARs) of NNIBP tolerant region II, various aromatic heterocyclic structures were also introduced into this region, yielding ten novel boronate-containing derivatives [20,21,22]. Additionally, the privileged 4-cyanovinyl-2,6-dimethylphenyl motif was also introduced to the left wing to develop additional π–π interactions with the highly conserved amino acid W229. The detailed SARs study of these derivatives and molecular docking simulation were also studied.

## 2. Results

### 2.1. Chemistry

**Scheme 1:**
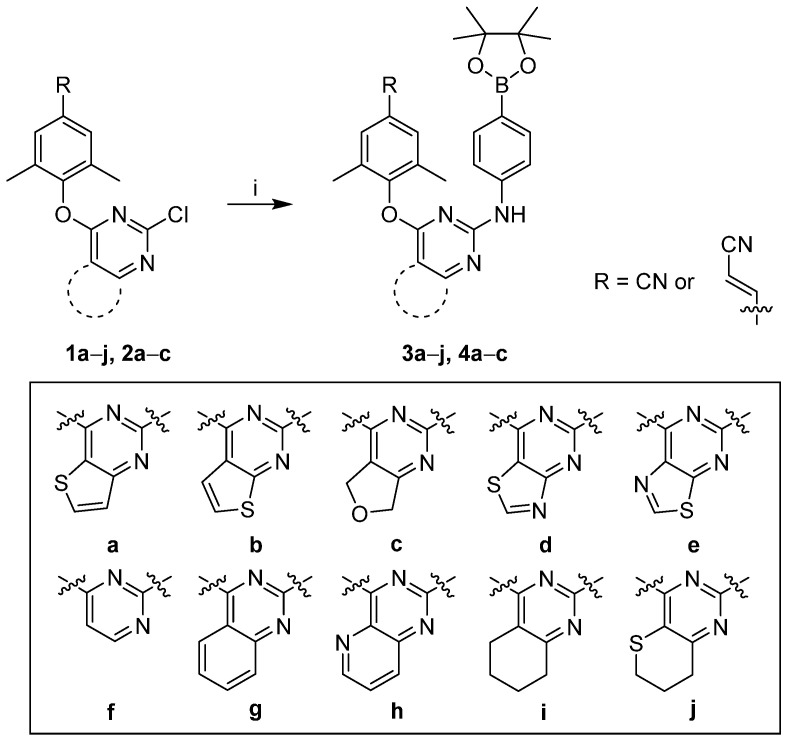
Synthesis of **3a**–**j** and **4a**–**c**. (Reagents and conditions: (i) 4-aminophenylboronic acid pinacol ester, Pd_2_(dba)_3_, BINAP, Cs_2_CO_3_, 1,4-dioxane, N_2_, 90 °C).

The synthetic protocols for the newly designed compounds **3a**–**j** and **4a**–**c** are outlined in Scheme 1. The synthetic routes and experimental procedures for **1a**–**j** and **2a**–**c** are described in more detail in the Supplementary Informations. All derivatives were prepared by well-established methods as described in our previous articles [20,21,23,24,25]. The previously prepared compounds **1a**–**j** and **2a**–**c** were selected as starting materials and reacted with 4-aminophenylboronic acid pinacol ester in the presence of BINAP and Pd_2_(dba)_3_ to yield the target compounds **3a**–**j** and **4a**–**c** via the Buchwald−Hartwig reaction. All novel target compounds were fully characterized by proton nuclear magnetic resonance spectroscopy (^1^H NMR) and carbon-13 nuclear magnetic resonance spectroscopy (^13^C NMR).

### 2.2. Anti-HIV-1 Activity Evaluation

All of the newly synthesized DAPY derivatives **3a**–**j** and **4a**–**c** were evaluated against the wild-type HIV-1 (IIIB) and the double-mutant strain RES056 (K103N/Y181C) in the MT-4 cell line using the 3-(4,5-dimethylthiazol-2-yl)-2,5-diphenyltetrazolium bromide (MTT) method. The approved NVP, EFV, and ETR were selected as control drugs. The values of EC_50_ (anti-HIV-1 potency), CC_50_ (cytotoxicity), and SI (selectivity index, CC_50_/EC_50_ ratio) of the target compounds are summarized in Table 1, Table 2 and Table 3.

As depicted in Table 1, the newly synthesized compounds exhibited moderate to high potency against the HIV-1 IIIB strain with EC_50_ values ranging from 0.009 to 6.17 μM. Among them, most of the target compounds displayed low sub-micromolar EC_50_ values ranging from 0.009 to 0.215 μM, being more potent or equipotent compared to that of NVP (EC_50_ = 0.151 μM). Unfortunately, all derivatives showed weaker efficacy or lost activity against the double-mutant strain RES056.

The anti-HIV-1 activities of the novel derivatives **3a**–**e** with the thiophene [3,2-d]pyrimidine (**3a**), thiophene [2,3-d]pyrimidine (**3b**), dihydrofuro-[3,4-d]pyrimidine (**3c**), thiazolo [4,5-d]-pyrimidine (**3d**), and thiazolo [5,4-d]-pyrimidine (**3e**) central cores demonstrated that the central scaffold has a great influence on the antiviral activity. When the thiophene [3,2-d]pyrimidine (**3a**, EC_50_ = 0.064 μM) was replaced by a dihydrofuro-[3,4-d]pyrimidine (**3c**, EC_50_ = 0.051 μM), the compound exhibited comparable antiviral activity, but the activity decreased sharply when the position of the sulfur atom was changed in the thiophene ring (**3b**, EC_50_ = 1.30 μM). Using quinazoline as the central core appeared to have a more negative effect on the potency (**3g**, EC_50_ = 6.17 μM). However, introducing a nitrogen atom at the C-8 position of the quinazoline core could significantly improve the activity (**3h**, EC_50_ = 0.112 μM), being 55-fold more potent than that of **3g**. Compound **3i**, with the 5,6,7,8-tetrahydroquinazoline core, displayed acceptable activity against HIV-1 IIIB (EC_50_ = 0.191 μM), and introducing sulfur atom at the C-8 position of the 5,6,7,8-tetrahydroquinazoline core led to a similar potency (**3j**, EC_50_ = 0.141 μM).

To design additional NNRTIs with improved activity against the mutant strain RES056, we replaced the cyano group in the left wing of **3a**–**c** with cyanovinyl motif, hoping that it would extend deeper into the hydrophobic channel and develop stronger interactions with the highly conserved residue W229. As shown in Table 1, all the derivatives (**4a**–**c**) exhibited increased potencies with EC_50_ values ranging from 0.009 μM to 0.014 μM. Among them, **4a** was turned out to be the most effective inhibitor against HIV-1 IIIB with an EC_50_ value of 0.009 μM, being much superior to that of NVP (EC_50_ = 0.151 μM) and comparable to those of ETR (EC_50_ = 0.004 μM) and EFV (EC_50_ = 0.003 μM). Moreover, **4a** displayed much lower cytotoxicity (CC_50_ > 238 μM), which was found to be up to 51-fold less than that of ETR (CC_50_ > 4.6 μM). For RES056 mutant strain, **4a** still showed no activity (EC_50_ > 238 μM), but **4b** and **4c** both exhibited better bioactivity (EC_50_ = 2.30 and 13.9 μM, respectively). Surprisingly, **4b** was 75-fold more potent than **3b** (EC50 = 2.30 vs. 174 μM). This proved that the substitution of cyano group for cyano vinyl group can indeed improve the biological activity of the compounds against RES056 strains.

Furthermore, **3a**, **3c**, and **4a**–**c** were selected to evaluate the ability to inhibit a variety of NNRTIs-resistant strains, including L100I, K103N, Y181C, Y188L, E138K, and F227L + V106A. As is depicted in Table 2, all the tested compounds maintained modest to high potencies against the whole mutant strains with EC_50_ values ranging from 0.005 to 9.10 μM, with the exception of **4a** in the F227L + V106A mutant strain. In the case of the K103N mutant strain, the most prevalent resistance to NVP and EFV, all these compounds displayed potent inhibitory activities with EC_50_ values ranging from 0.005 to 0.120 μM, being superior or comparable to that of NVP (EC_50_ = 3.93 μM). Particularly, compounds **4a** (EC_50_ = 0.005 μM) and **4b** (EC_50_ = 0.008 μM) provided the highest potencies towards K103N, which were far superior to those of NVP and EFV (EC_50_ = 0.632 and 0.071 μM, respectively) and were comparable to those of ETR (EC_50_ = 0.003 μM). Moreover, **4a** also maintained high potency against L100I mutant strain with EC_50_ value of 0.186 μM, being 3.3-fold more potent than that of NVP (EC_50_ = 0.623 μM). More interestingly, all these selected compounds displayed highly potent inhibitory activities against the E138K mutant strain (EC_50_ = 0.021–0.193 μM), among which **4a** (EC_50_ = 0.021 μM) and **4b** (EC_50_ = 0.030 μM) were the most potent derivatives and exhibited higher activities compared to those of NVP (EC_50_ = 0.168 μM). Moreover, **4a** also exhibited the most potent inhibitory activity against the Y181C mutant strain (EC_50_ = 1.61 μM), being superior to that of NVP (EC_50_ = 5.06 μM). It is worth noting that compounds **3a** and **4b** (EC_50_ = 4.95 and 3.21 μM, respectively) showed the prominent inhibitory activities against the F227L + V106A strain, which were superior to NVP (EC_50_ = 8.05 μM). In addition, the selectivity index (SI) and resistance fold (RF) of the tested compounds were summarized in Table 3.

### 2.3. HIV-1 RT Inhibition Assay

To validate the binding target of these derivatives, **3a**, **3c**, and **4a**–**c** were selected to test their inhibitory activity against the recombinant wild-type HIV-1 RT enzyme. As displayed in Table 4, most compounds showed high binding-affinity with the wild-type HIV-1 RT (IC_50_ = 0.047–0.354 μM). The most potent compound **4a** exhibited the highest RT inhibitory activity (IC_50_ = 0.047 μM), being much more potent than that of NVP (IC_50_ = 0.638 μM) and comparable to that of ETR (IC_50_ = 0.011 μM). The preliminary results demonstrated that these newly synthesized derivatives could bind to HIV-1 RT and behave as typical NNRTIs.

### 2.4. Molecular Docking (MD) Simulation

In order to verify the binding interactions of these newly synthesized compounds and to obtain further insights into their binding modes in the NNIBP of RT, 4a was selected as the representative compound in further molecular docking studies. The co-crystal structures of HIV-1 WT RT/K-5a2 (PDB code: 6C0J) and HIV-1 K103N/Y181C mutant RT/25a (PDB code: 6C0R) were used as the input structure for docking calculation (K-5a2 and 25a were two potent diarypyrimidine (DAPY) derivatives with similar center core to **4a** found in our previous work [21,25]). PyMOL was used to visualize the results. The docking protocol is described in the experimental section.

**Figure 3:**
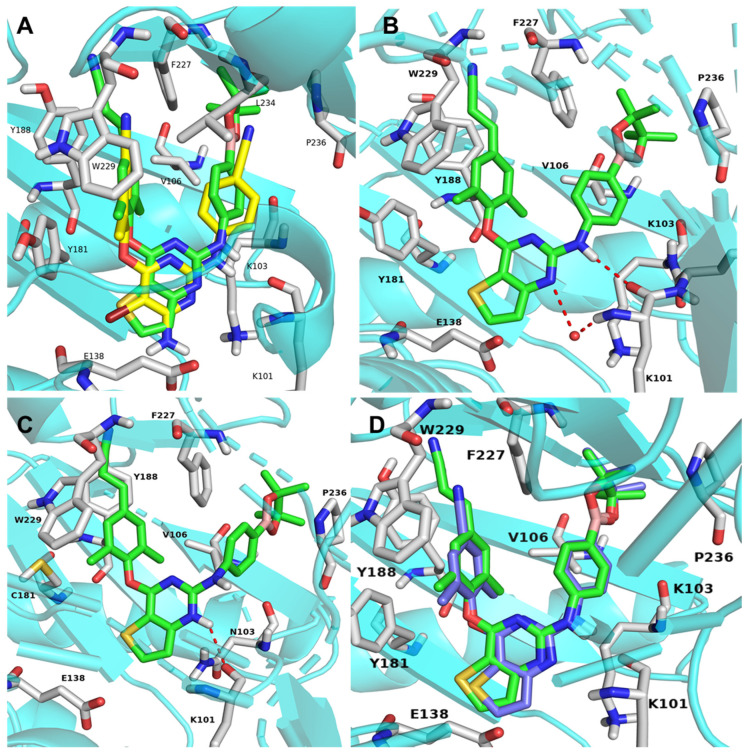
(**A**) The overlapping conformations of **4a** with the lead compound ETR (green, **4a**; yellow, ETR). (**B**) Predicted binding modes of **4a** with HIV-1 WT RT (PDB code: 6C0J; green, **4a**); (**C**) Predicted binding modes of **4a** with HIV-1 K103N/Y181C mutant RT (PDB code: 6C0R). (**D**) Overlapping patterns of **3a** with **4a** (green, **4a**; slate, **3a**). The hydrogen bonds between the inhibitors and amino acid residues are indicated with dashed lines (red). Nonpolar-hydrogen atoms are omitted for clarity.

The binding mode of **4a** with HIV-1 WT RT is shown in Figure 3. The key amino acid residues surrounding the ligand appear as light gray sticks. The overlapping conformations of **4a** with the lead compound ETR is shown in Figure 3A. The results indicated that **4a** binds with NNIBP in a horseshoe-like conformation, which is similar to that seen with NNRTIs of the DAPY family, and fully occupies the cavity in NNIBP and remains adaptable to adapt to the cavity due to its high flexibility. As shown in Figure 3B, detailed binding interactions reveal the following features. First, the left 4-cyanovinyl-2,6-dimethylphenyl group of **4a** fully occupies the hydrophobic cavity surrounded by hydrophobic aromatic amino acid residues Y181, Y188, F227 and W229, exhibiting a π−π interaction with these residues. Second, the N-atom of the center core and NH linker connecting the central pyrimidine ring and the right wing are involved in double hydrogen bonding with the backbone of K101 through water bridges or directly, which are conserved hydrogen bonds among the second-generation NNRTIs/RT complexes. Moreover, the phenyl-linked 4,4,5,5-tetramethyl-1,3,2-dioxaborolane is directed to the tolerant region I and can develop extensive interactions with surrounding lipophilic amino acids. In regard to RT carrying the K103N/Y181C double-mutation, Y181C mutation abolishes the favorable π−π stacking interactions between the Y181 side chain and the 4-cyanovinyl-2,6-dimethylphenyl group of **4a**, which greatly reduces the binding interface between **4a** and C181. Additionally, the dramatic changes in NNIBP result in a decrease of the buried interface between **4a** and residue 103, which leads to the lack of the extensive hydrogen-bonding network between **4a** and the backbone of K101 (Figure 3C). The overlapping conformations of **3a** and **4a** are shown in Figure 3D. The cyanovinyl group of **4a** is able to reach deeper into the hydrophobic pocket than the cyano group of **3a** and forms a π−π interaction with surrounding hydrophobic amino acid residues, such as F227 and W229. This may have contributed to the superior biological activity of **4a** compared to that of **3a**.

### 2.5. In Silico Prediction of Physicochemical Properties

The drug-like properties of representative compounds **4a**, **4b** and control drugs ETR and RPV were characterized using a free online software ADMETlab 2.0 (https://admetmesh.scbdd.com/, accessed on 30 September 2022). As shown in Table 5, the results indicated that the various parameters of **4a** and **4b**, including hydrogen bond acceptors (nHA), hydrogen bond donors (nHD), topological polar surface area (tPSA), and rotatable bonds (nRot) were all in the optimal range. However, due to the presence of multiple aromatic rings and cyclic borate ester structure in **4a** and **4b**, their LogP values are higher than those of ETR and RPV.

## 3. Materials and Methods

### 3.1. Synthesis

All melting points were determined on a micro melting point apparatus (RY-1G, Tianjin Tian Guang Optical Instruments). ^1^H NMR and ^13^C NMR spectra were recorded in DMSO-d_6_ on a Bruker AV-400 spectrometer with tetramethylsilane (TMS) as the internal standard; signals are abbreviated as s (singlet), d (doublet), t (triplet), and m (multiplet). Chemical shifts are reported in δ values (ppm) from TMS and coupling constants are given in hertz (Hz). The mass spectra were measured in AG1313A Standard LC Autosampler (Agilent). All reactions were routinely monitored by thin layer chromatography (TLC) on Silica Gel GF254 for TLC (Merck) and spots were visualized with iodine vapor or by irradiation with UV light (λ = 254 and 356 nm). Flash column chromatography was performed on columns packed with Silica Gel (Qingdao Haiyang Chemical Company). Solvents were purified and dried by standard methods. The concentration of the reaction solutions involved the use of rotary evaporator at reduced pressure.

#### 3.1.1. General Procedure for the Preparation of Target Compounds **3a**–**j** and **4a**–**c**

##### 3,5-Dimethyl-4-((2-((4-(4,4,5,5-tetramethyl-1,3,2-dioxaborolan-2-yl)phenyl)amino) thieno [3,2-d]pyrimidin-4-yl)oxy)benzonitrile (**3a**) (72%, yield)

A reaction mixture of Pd_2_(dba)_3_ (0.072 g, 0.079 mmol) and BINAP (0.05 g, 0.079 mmol) in 10 mL of dry dioxane was stirred at room temperature for 10 min and then 4-aminophenylboronic acid pinacol ester (0.416 g, 1.9 mmol) and Cs_2_CO_3_ (0.323 g, 2.38 mmol) were added. Compound **1a** (0.5 g, 1.58 mmol) was added to the mixture after additional 10 min, and then the flask was evacuated and backfilled with nitrogen. The mixture was stirred at 90 °C for an extra 8 h. Then the solvent was evaporated under reduced pressure, and the obtained residue was dissolved in 20 mL of ethyl acetate (EA). The organic phase was washed with saturated sodium chloride (35 mL) and then dried over anhydrous Na_2_SO_4_, filtered, concentrated to dryness by rotary evaporation, and purified by flash column chromatography to give the target compound **3a** (72%). White powder. ^1^H NMR (400 MHz, DMSO-d_6_) δ 9.72 (s, 1H), 8.37 (d, J = 5.3 Hz, 1H), 7.82 (s, 2H), 7.46 (d, J = 8.2 Hz, 2H), 7.43 (d, J = 5.3 Hz, 1H), 7.40 (d, J = 8.2 Hz, 2H), 2.16 (s, 6H), 1.28 (s, 12H) ppm. ^13^C NMR (100 MHz, DMSO-d_6_) δ 165.4, 162.6, 157.9, 153.6, 143.8, 138.1, 135.3, 133.3, 133.2, 123.9, 118.9, 117.7, 109.4, 107.6, 83.7, 25.2, 16.2 ppm. HPLC 99.15%. mp: 168–170 °C. C_27_H_27_BN_4_O_3_S (498.2).

The synthetic procedures for target compounds **3b**–**c** and **4a**–**c** were similar to that of **3a**.

##### 3,5-Dimethyl-4-((2-((4-(4,4,5,5-tetramethyl-1,3,2-dioxaborolan-2-yl)phenyl)amino) thieno [2,3-d]pyrimidin-4-yl)oxy)benzonitrile (**3b**) (65%, yield)

White powder. ^1^H NMR (400 MHz, DMSO-d_6_) δ 9.90 (s, 1H), 7.77 (s, 2H), 7.45 (d, J = 5.8 Hz, 1H), 7.40 (d, J = 5.8 Hz, 1H), 7.16 (d, J = 8.3 Hz, 2H), 6.50 (d, J = 8.3 Hz, 2H), 2.15 (s, 6H), 1.27 (s, 12H) ppm. ^13^C NMR (100 MHz, DMSO-d_6_) δ 162.2, 160.6, 157.0, 152.7, 150.5, 147.9, 133.2, 133.0, 132.1, 120.7, 119.1, 118.7, 115.2, 109.0, 83.8, 25.4, 16.3 ppm. HPLC 97.22%. mp: 142–144 °C. C_27_H_27_BN_4_O_3_S (498.2).

##### 3,5-Dimethyl-4-((2-((4-(4,4,5,5-tetramethyl-1,3,2-dioxaborolan-2-yl)phenyl)amino) -5,7-dihydrofuro [3,4-d]pyrimidin-4-yl)oxy)benzonitrile (**3c**) (62%, yield)

White powder. ^1^H NMR (400 MHz, DMSO-d_6_) δ 9.90 (s, 1H), 7.79 (s, 2H), 7.36 (d, J = 8.3 Hz, 2H), 7.29 (d, J = 8.3 Hz, 2H), 5.11 (s, 2H), 4.92 (s, 2H), 2.14 (s, 6H), 1.27 (s, 12H) ppm. ^13^C NMR (100 MHz, DMSO-d_6_) δ 174.3, 162.4, 160.4, 153.8, 143.4, 135.3, 133.2, 133.0, 128.6, 119.0, 117.7, 109.1, 105.3, 83.7, 72.3, 69.5, 25.1, 16.2 ppm. HPLC 97.86%. mp: 149–151 °C. C_27_H_29_BN_4_O_4_ (484.2).

##### 3,5-Dimethyl-4-((5-((4-(4,4,5,5-tetramethyl-1,3,2-dioxaborolan-2-yl)phenyl)amino) thiazolo [4,5-d]pyrimidin-7-yl)oxy)benzonitrile (**3d**) (72%, yield)

White powder. ^1^H NMR (400 MHz, DMSO-d_6_) δ 9.01 (s, 1H), 8.90 (s, 1H), 7.76 (s, 2H), 7.15 (d, J = 8.3 Hz, 2H), 6.51 (d, J = 8.3 Hz, 2H), 2.17 (s, 6H), 1.27 (s, 12H) ppm. ^13^C NMR (100 MHz, DMSO-d_6_) δ 162.2, 160.6, 157.0, 153.6, 152.7, 150.5, 147.9, 133.2, 120.7, 119.1, 118.7, 115.2, 109.0, 83.8, 25.4, 16.3 ppm. HPLC 97.59%. mp: 117–119 °C.C_26_H_26_BN_5_O_3_S (499.2).

##### 3,5-Dimethyl-4-((5-((4-(4,4,5,5-tetramethyl-1,3,2-dioxaborolan-2-yl)phenyl)amino) thiazolo [5,4-d]pyrimidin-7-yl)oxy)benzonitrile (**3e**) (66%, yield)

White powder. ^1^H NMR (400 MHz, DMSO-d_6_) δ 9.04 (s, 1H), 8.92 (s, 1H), 7.77 (s, 2H), 7.16 (d, J = 8.3 Hz, 2H), 6.50 (d, J = 8.3 Hz, 2H), 2.15 (s, 6H), 1.27 (s, 12H) ppm. ^13^C NMR (100 MHz, DMSO-d_6_) δ 162.2, 160.6, 157.0, 153.6, 152.7, 150.5, 147.9, 133.2, 120.7, 119.1, 118.7, 115.2, 109.0, 83.8, 25.4, 16.3 ppm. HPLC 99.00%. mp: 165–167 °C. C_26_H_26_BN_5_O_3_S (499.2).

##### 3,5-Dimethyl-4-((2-((4-(4,4,5,5-tetramethyl-1,3,2-dioxaborolan-2-yl)phenyl)amino) pyrimidin-4-yl)oxy)benzonitrile (**3f**) (75%, yield)

White powder. ^1^H NMR (400 MHz, DMSO-d_6_) δ 9.83 (s, 1H), 8.46 (d, J = 5.5 Hz, 1H), 7.78 (s, 2H), 7.40 (s, 4H), 6.62 (d, J = 5.5 Hz, 1H), 2.14 (s, 6H), 1.28 (s, 12H) ppm. ^13^C NMR (100 MHz, DMSO-d_6_) δ 168.3, 161.0, 159.9, 153.9, 143.4, 135.3, 133.2, 133.0, 119.0, 117.8, 108.9, 98.3, 83.7, 25.1, 16.2 ppm. HPLC 96.00%. mp: 202–204 °C. C_25_H_27_BN_4_O_3_ (442.2).

##### 3,5-Dimethyl-4-((2-((4-(4,4,5,5-tetramethyl-1,3,2-dioxaborolan-2-yl)phenyl)amino) quinazolin-4-yl)oxy)benzonitrile (**3g**) (60%, yield)

White powder. ^1^H NMR (400 MHz, DMSO-d_6_) δ 9.01 (s, 1H), 8.36–8.11 (m, 1H), 7.78 (s, 2H), 8.04–7.53 (m, 3H), 7.52–7.29 (m, 4H), 2.16 (s, 6H), 1.28 (s, 12H) ppm. ^13^C NMR (100 MHz, DMSO-d_6_) δ 165.7, 165.5, 156.3, 153.9, 152.7, 135.3, 135.2, 133.1, 133.1, 132.4, 129.4, 129.0, 128.7, 125.5, 124.0, 123.3, 122.3, 120.9, 118.1, 115.2, 109.2, 109.1, 83.7, 25.2, 16.3, 16.3 ppm. HPLC 98.60%. mp: 147–149 °C. C_29_H_29_BN_4_O_3_ (492.2).

##### 3,5-Dimethyl-4-((2-((4-(4,4,5,5-tetramethyl-1,3,2-dioxaborolan-2-yl)phenyl)amino) pyrido [3,2-d]pyrimidin-4-yl)oxy)benzonitrile (**3h**) (52%, yield)

White powder. ^1^H NMR (400 MHz, DMSO-d_6_) δ 9.92 (s, 1H), 8.97 (dd, J = 4.5, 2.0 Hz, 1H), 8.59 (dd, J = 8.5, 2.1 Hz, 1H), 7.74 (s, 2H), 7.69–7.61 (m, 2H), 7.43 (d, J = 7.2 Hz, 2H), 7.39 (dd, J = 8.8, 5.0 Hz, 1H), 2.11 (s, 6H), 1.21 (s, 12H) ppm. ^13^C NMR (100 MHz, DMSO-d_6_) δ 168.9, 162.0, 161.8, 161.3, 159.0, 158.4, 157.9, 153.7, 153.5, 153.1, 143.2, 135.3, 134.3, 133.7, 133.2, 133.1, 132.8, 122.6, 120.2, 119.0, 118.8, 118.6, 109.4, 108.9, 108.1, 106.3, 83.8, 25.2, 16.3 ppm. HPLC 96.32%. mp: 177–179 °C. C_28_H_28_BN_5_O_3_ (493.2).

##### 3,5-Dimethyl-4-((2-((4-(4,4,5,5-tetramethyl-1,3,2-dioxaborolan-2-yl)phenyl)amino) -5,6,7,8-tetrahydroquinazolin-4-yl)oxy)benzonitrile (**3i**) (63%, yield)

White powder. ^1^H NMR (400 MHz, DMSO-d_6_) δ 8.92 (s, 1H), 7.72 (s, 2H), 7.05 (d, J = 8.5 Hz, 2H), 6.42 (d, J = 8.5 Hz, 2H), 2.81–2.56 (m, 4H), 2.10 (s, 6H), 1.92–1.71 (m, 4H), 1.27 (s, 12H) ppm. ^13^C NMR (100 MHz, DMSO-d_6_) δ 168.1, 166.0, 157.4, 154.8, 151.9, 133.2, 132.9, 132.7, 119.7, 115.0, 108.4, 104.7, 31.9, 25.3, 22.4, 22.4, 21.4, 16.2 ppm. HPLC 99.51%. mp: 233–235 °C. C_29_H_33_BN_4_O_3_ (496.3).

##### 3,5-Dimethyl-4-((2-((4-(4,4,5,5-tetramethyl-1,3,2-dioxaborolan-2-yl)phenyl)amino) -7,8-dihydro-6H-thiopyrano [3,2-d]pyrimidin-4-yl)oxy)benzonitrile (**3j**) (59%, yield)

White powder. ^1^H NMR (400 MHz, DMSO-d_6_) δ 8.85 (s, 1H), 7.66 (s, 2H), 6.95 (d, J = 8.4 Hz, 2H), 6.34 (d, J = 8.4 Hz, 2H), 3.01 (t, J = 6.3 Hz, 2H), 2.72 (t, J = 6.3 Hz, 2H), 2.10 (p, J = 6.3 Hz, 2H), 2.02 (s, 6H), 1.28 (s, 12H) ppm. ^13^C NMR (100 MHz, DMSO-d_6_) δ 163.4, 163.1, 155.8, 154.4, 152.1, 133.1, 132.9, 132.6, 119.8, 115.1, 108.8, 100.8, 31.7, 26.3, 25.3, 23.3, 16.2 ppm. HPLC 100.00%. mp: 201–203 °C. C_28_H_31_BN_4_O_3_S (514.2).

##### (E)-3-(3,5-Dimethyl-4-((2-((4-(4,4,5,5-tetramethyl-1,3,2-dioxaborolan-2-yl)phenyl) amino)thieno [3,2-d]pyrimidin-4-yl)oxy)phenyl)acrylonitrile (**4a**) (72%, yield)

White powder. ^1^H NMR (400 MHz, DMSO-d_6_) δ 9.75 (s, 1H), 8.36 (d, J = 5.4 Hz, 1H), 7.69 (d, J = 16.7 Hz, 1H), 7.60 (s, 2H), 7.42 (d, J = 5.4 Hz, 1H), 7.39 (d, J = 8.3 Hz, 2H), 7.33 (d, J = 8.3 Hz, 2H), 6.51 (d, J = 16.7 Hz, 1H), 2.12 (s, 6H), 1.28 (s, 12H) ppm. ^13^C NMR (100 MHz, DMSO-d_6_) δ 165.3, 163.1, 157.9, 152.0, 150.3, 143.9, 137.8, 135.3, 132.2, 131.9, 128.8, 123.8, 119.3, 117.6, 107.5, 97.1, 83.7, 25.1, 16.4 ppm. HPLC 98.84%. mp: 231–233 °C. C_29_H_29_BN_4_O_3_S (524.2).

##### (E)-3-(3,5-Dimethyl-4-((2-((4-(4,4,5,5-tetramethyl-1,3,2-dioxaborolan-2-yl)phenyl) amino)thieno [2,3-d]pyrimidin-4-yl)oxy)phenyl)acrylonitrile (**4b**) (70%, yield)

White powder. ^1^H NMR (400 MHz, DMSO-d_6_) δ 9.75 (s, 1H), 8.35 (d, J = 5.4 Hz, 1H), 7.69 (d, J = 16.7 Hz, 1H), 7.60 (s, 2H), 7.41 (d, J = 5.4 Hz, 1H), 7.38 (d, J = 8.3 Hz, 2H), 7.32 (d, J = 8.3 Hz, 2H), 6.51 (d, J = 16.7 Hz, 1H), 2.12 (s, 6H), 1.28 (s, 12H) ppm. ^13^C NMR (100 MHz, DMSO-d_6_) δ 165.3, 163.1, 157.9, 152.0, 150.3, 143.9, 137.8, 135.3, 132.2, 131.9, 128.8, 123.8, 119.3, 117.6, 107.5, 97.1, 83.7, 25.1, 16.4 ppm. HPLC 99.18%. mp: 263–265 °C. C_29_H_29_BN_4_O_3_S (524.2).

##### (E)-3-(3,5-Dimethyl-4-((2-((4-(4,4,5,5-tetramethyl-1,3,2-dioxaborolan-2-yl)phenyl) amino)-5,7-dihydrofuro [3,4-d]pyrimidin-4-yl)oxy)phenyl)acrylonitrile (**4c**) (66%, yield)

White powder. ^1^H NMR (400 MHz, DMSO-d_6_) δ 9.82 (s, 1H), 7.62 (d, J = 16.7 Hz, 1H), 7.51 (s, 2H), 7.19 (d, J = 8.4 Hz, 2H), 7.15 (d, J = 8.4 Hz, 2H), 6.43 (d, J = 16.7 Hz, 1H), 5.03 (s, 2H), 4.84 (s, 2H), 2.03 (s, 6H), 1.20 (s, 12H) ppm. ^13^C NMR (100 MHz, DMSO-d_6_) δ 160.4, 160.0, 155.6, 150.0, 144.6, 138.7, 134.1, 132.8, 130.9, 129.9, 129.9, 120.9, 120.4, 118.3, 98.7, 84.2, 73.0, 72.4, 24.7, 16.4 ppm. HPLC 98.01%. mp: 134–136 °C. C_29_H_31_BN_4_O_4_ (510.2).

### 3.2. In Vitro Anti-HIV Activities Assays in MT-4 Cells

The 3-(4,5-dimethylthiazol-2-yl)-2,5-diphenyltetrazolium bromide (MTT) method was used to evaluate the antiviral activity and cytotoxicity of the synthesized compounds as previously described [26,27]. At the beginning of each experiment, stock solutions (10× final concentration) of test compounds were added in 25 μL volumes to two series of triplicate wells for allowing the simultaneous evaluation of their effects on mock- and HIV-infected cells. The serial 5-fold dilutions of the compounds were made in flat-bottomed 96-well microtiter trays directly, including untreated control HIV-1 and mock infected cell samples for each sample using a Biomek 3000 robot (Beckman Instruments, Fullerton, CA, USA). HIV-1 (IIIB) and mutant HIV-1 strains (RES056, F227L/V106A, L100I, K103N, E138K, Y181C, and Y188L) stock (50 μL at 100–300 CCID_50_) (50% cell culture infectious dose) or culture medium was added to either the infected or mock-infected wells of the microtiter tray. Mock-infected cells were used to evaluate the effect on uninfected cells to assess the cytotoxicity of the test compounds. Exponentially growing MT-4 cells were centrifuged for 5× *g* min at 1000 rpm (Eppendorf 5424, Hamburg, Germany) and then the supernatant was discarded. The MT-4 cells were resuspended at 6 × 105 cells/mL and 50 μL aliquots were transferred to the microtiter tray wells. At 5 days after infection, the viability of mock- and HIV-infected cells was determined spectrophotometrically by means of MTT assay.

The MTT assay is based on the reduction of yellow-colored MTT (Acros Organics, Geel, Belgium) by mitochondrial dehydrogenase of metabolically active cells to form a blue-purple formazan. The absorbances were read in an eight-channel computer-controlled photometer at the wavelengths of 540 and 690 nm. All data were calculated using the median optical density (OD) value of three wells. The 50% effective antiviral concentration (EC_50_) was defined as the concentration of the test compound affording 50% protection from viral cytopathogenicity. The CC_50_ was defined as the compound concentration that reduced the absorbance (OD_540_) of mock-infected cells by 50%.

### 3.3. HIV-1 RT Inhibition Assays

The HIV-1 RT inhibition assay was performed by using an RT assay kit produced by Roche (Basel, Switzerland). The procedure for assaying HIV-1 RT inhibition was conducted as the kit protocol [28].

First, the HIV-1 RT enzyme, reconstituted template, and viral nucleotides (digoxigenin (DIG)-dUTP, biotin-dUTP, and dTTP) were incubated for 1 h at 37 °C in the incubation buffer with or without inhibitors. Then the reaction mixture was transferred to a streptavidin-coated microtiter plate (MTP) and incubated for another 1 h at 37 °C. The biotin-labeled dNTPs were incorporated into the cDNA chain in the presence of RT and bound to streptavidin. The washing buffer was used to wash the unbound dNTPs and add the anti-DIG-POD to the MTPs.

After incubation for 1 h at 37 °C, the DIG-labeled dNTPs incorporated in cDNA were bound to the anti-DIG-POD antibody. The peroxide substrate (ABST) solution was added to the MTPs, when the unbound anti-DIG-PODs were washed out. The reaction mixture was incubated at 25 °C until the green color was sufficiently developed for detection. The absorbance of the sample was determined at OD_405_ nm using a microtiter plate ELISA reader. The percentage inhibitory activity of RT inhibitors was calculated according to the following formula: % inhibition = [O.D. value with RT but without inhibitors − O.D. value with RT and inhibitors]/[O.D. value with RT and inhibitors − O.D. value without RT and inhibitors]. The IC_50_ values correspond to the concentrations of the inhibitors required to inhibit biotin-dUTP incorporation by 50%.

### 3.4. Molecular Simulations

Molecular docking was performed by using the Schrödinger 2019-1. The Protein Preparation Wizard tool of Maestro was used to prepare the protein structure required for subsequent docking calculations [29]. The structure of protein was minimized using OPLS4 force field [30] to optimize hydrogen bonding network. The receptor grid generation tool in Maestro was employed to define an active site around the native ligand to cover all the residues within 20 Å from it. The compound **4a** were created virtually through the Maestro 2D Sketcher tool. Then, using Ligprep tool to prepare the ligand for the docking experiments. Then, the ligands were docked using a ligand docking tool [31]. All parameters in the preceding operations were default settings and the best-ranked compounds were retained for subsequent analysis.

## 4. Conclusions

In this study, a series of novel boronate-containing DAPY derivatives were designed, synthesized, and evaluated for their anti-HIV-1 potencies. Most of the synthesized compounds exhibited significant inhibitory activities (EC_50_ < 6.17 μM) towards HIV-1 IIIB strain in MT-4 cells. Among them, **3a**, **3c**, and **4a**–**c** displayed robust potencies (EC_50_ = 0.009–0.064 μM), which were more potent than that of NVP (EC_50_ = 0.151 μM). As for HIV-1 RT mutant strains, most of the tested compounds exhibited excellent potencies. Notably, **4a** was a highly potent inhibitor against HIV-1 mutant strains with EC_50_ values of 0.186 μM (L100I), 0.005 μM (K103N), 0.648 μM (Y188C), and 0.021 μM (E138K), respectively. In addition, **4a** has much lower cytotoxicity (CC_50_ > 238 μM) and a higher SI value of 27516. Moreover, **4b** also turned out to be an effective inhibitor against the double-mutant strains F227L + V106A and RES056 with prominent EC_50_ values of 3.21 and 2.30 μM, respectively. The HIV-1 RT enzyme inhibitory activity assay suggested that **4a** (IC_50_ = 0.167 μM) acted as a typical NNRTIs. The molecular docking study was also investigated and gave a reasonable explanation of the preliminary SARs. Consequently, compound **4a** showed the highest activity and low cytotoxicity and could be used as a lead for further modifications to obtain more potent boronate-containing NNRTIs.

## Figures and Tables

**Table 1:**
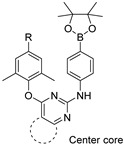
Activity against HIV-1 IIIB and RES056, cytotoxicity, and SI values of **3a**–**j** and **4a**–**c**.

Compds.	R	Center Core	EC_50_ (μM) *^a^*		CC_50_ (μM) *^b^*	SI *^c^* (RF) *^d^*	
			IIIB	RES056		IIIB	RES056
**3a**	CN	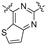	0.064 ± 0.021	>27.4	27.4 ± 1.20	431 (1.0)	<1 (>431)
**3b**	CN	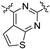	1.30 ± 0.275	174 ± 34.5	>254	>193 (1.0)	>1 (134)
**3c**	CN	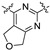	0.051 ± 0.015	25.8 ± 7.93	94.8 ± 32.2	1847 (1.0)	<1 (503)
**3d**	CN	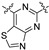	0.120 ± 0.056	>24.1	24.1 ± 3.76	201 (1.0)	<1 (>201)
**3e**	CN	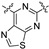	0.215 ± 0.091	>26.5	26.5 ± 1.78	123 (1.0)	<1 (>123)
**3f**	CN	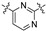	0.149 ± 0.049	>30.6	30.6 ± 1.92	206 (1.0)	<1 (>206)
**3g**	CN	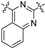	6.17 ± 1.83	>254	>254	>41 (1.0)	X1 *^e^* (>41.1)
**3h**	CN	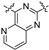	0.112 ± 0.024	>26.0	26.0 ± 2.33	232 (1.0)	<1 (>232)
**3i**	CN	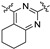	0.191 ± 0.088	>27.7	27.7 ± 1.27	145 (1.0)	<1 (>145)
**3j**	CN	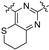	0.141 ± 0.053	>25.9	25.9 ± 1.41	184 (1.0)	<1 (>184)
**4a**	CV	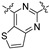	0.009 ± 0.002	>238	>238	>27,516 (1.0)	X1 *^e^* (>27516)
**4b**	CV	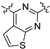	0.010 ± 0.003	2.30 ± 0.660	22.1 ± 3.69	2161 (1.0)	10 (224)
**4c**	CV	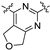	0.014 ± 0.006	13.9 ± 9.32	80.3 ± 31.3	5890 (1.0)	6 (1018)
**NVP**	-	-	0.151 ± 0.037	>15.0	>15.0	>100 (1.0)	X1 *^e^* (99.9)
**EFV**	-	-	0.003 ± 0.001	0.337 ± 0.149	>6.35	>2268 (1.0)	>19 (120)
**ETR**	-	-	0.004 ± 0.001	0.061 ± 0.034	>4.60	>1105 (1.0)	>76 (14.6)

*^a^* EC_50_: concentration of compound required to achieve 50% protection of MT-4 cell cultures against HIV-1-induced cytopathicity, as determined using the MTT method. *^b^* CC_50_: concentration required to reduce the viability of mock-infected cell cultures by 50%, as determined using the MTT method. *^c^* SI: selectivity index, the ratio of CC_50_/EC_50_. *^d^* RF is the ratio of EC_50(resistant strain)_/EC_50(wild-type strain)_. *^e^* X1: X1 means a value of SI > 1 or SI ≤ 1.

**Table 2:** Activity of target compounds against mutant HIV-1 strains.

Compds.	EC_50_ (μM) *^a^*					
	L100I	K103N	Y181C	Y188L	E138K	F227L + V106A
**3a**	2.51 ± 0.155	0.081 ± 0.008	6.29 ± 3.27	6.02 ± 3.14	0.193 ± 0.077	4.95 ± 1.98
**3c**	2.43 ± 0.302	0.120 ± 0.019	4.37 ± 2.19	4.52 ± 1.54	0.130 ± 0.041	9.10 ± 2.64
**4a**	0.186 ± 0.032	0.005 ± 0.006	0.648 ± 0.162	1.61 ± 0.508	0.021 ± 0.007	>1.81
**4b**	0.280 ± 0.055	0.008 ± 0.002	0.675 ± 0.188	2.52 ± 0.238	0.030 ± 0.009	3.21 ± 1.97
**4c**	0.553 ± 0.152	0.026 ± 0.005	1.28 ± 0.489	3.96 ± 0.894	0.039 ± 0.018	5.88 ± 2.52
**NVP**	0.623 ± 0.277	3.93 ± 1.01	5.06 ± 0.922	9.08 ± 2.41	0.168 ± 1.077	8.05 ± 3.54
**EFV**	0.032 ± 0.008	0.071 ± 0.020	0.005 ± 0.002	0.261 ± 0.118	0.006 ± 0.002	0.381 ± 0.249
**ETR**	0.008 ± 0.002	0.003 ± 0.008	0.016 ± 0.005	0.019 ± 0.007	0.010 ± 0.005	0.026 ± 0.017

*^a^* EC_50_: concentration of compound required to achieve 50% protection of MT-4 cell cultures against HIV-1-induced cytopathicity, as determined using the MTT method.

**Table 3:** SI and RF values of representative compounds.

Compds.	SI *^a^* (RF) *^b^*					
	L100I	K103N	Y181C	Y188L	E138K	F227L + V106A
**3a**	11 (39.4)	341 (1.3)	4 (98.8)	5 (94.7)	142 (3.0)	6 (77.8)
**3c**	39 (47.3)	792 (2.3)	22 (85.1)	21 (87.9)	731 (2.5)	10 (177.2)
**4a**	>1285 (21.4)	>47,170 (0.6)	>368 (74.8)	>148 (506.8)	>11,261 (2.4)	X1 *^c^* (209.7)
**4b**	79 (27.4)	2866 (0.8)	33 (65.9)	9 (246.1)	732 (3.0)	7 (313.5)
**4c**	145 (40.6)	3110 (1.9)	63 (94.2)	20 (290.3)	2036 (2.9)	14 (431.5)
**NVP**	>24 (4.1)	>4 (26.1)	>3 (33.6)	>2 (60.4)	>89 (1.1)	>2 (53.5)
**EFV**	>200 (11.3)	>89 (25.4)	>1260 (1.8)	>24 (93.1)	>1149 (2.0)	>17 (136.2)
**ETR**	>549 (2.0)	>1417 (0.8)	>297 (3.7)	>248 (4.5)	>469 (2.4)	>178 (6.2)

*^a^* SI: selectivity index, the ratio of CC_50_/EC_50_. *^b^* RF is the ratio of EC_50_ (resistant viral strain)/EC_50_ (wild-type viral strain). *^c^* X1: X1 means a value of SI > 1 or SI ≤ 1.

**Table 4:** Inhibitory activity against WT HIV-1 RT of representative compounds.

Compds.	IC_50_ (μM) *^a^*	Compds.	IC_50_ (μM) *^a^*
**3a**	0.354 ± 0.043	**4c**	0.200 ± 0.028
**3c**	0.228 ± 0.045	**NVP**	0.638 ± 0.269
**4a**	0.167 ± 0.031	**EFV**	0.008 ± 0.003
**4b**	0.047 ± 0.001	**ETR *^b^***	0.011 ± 0.000

*^a^* IC_50_: inhibitory concentration of test compounds required to inhibit biotin deoxyuridine triphosphate (biotin-dUTP) incorporation into WT HIV-1 RT by 50%. *^b^* See ref. [21].

**Table 5:** Inhibitory activity against WT HIV-1 RT of representative compounds.

Compds.	nHA	nHD	TPSA	nRot	LogP
**4a**	**7**	**1**	**92.52**	**5**	**6.549**
**4b**	**7**	**1**	**92.52**	**5**	**6.707**
**ETR**	**7**	**3**	**124.6**	**3**	**3.47**
**RPV**	**6**	**2**	**103.88**	**3**	**3.47**
**Optimal range**	**0–12**	**0–7**	**0–140**	**0–11**	**0–3**

nHA = number of hydrogen bond acceptors; nHD = number of hydrogen bond donors; nRot = number of rotatable bonds; TPSA = topological polar surface area; LogP = predicted Log P.

## Data Availability

Not applicable.

## Supplementary Materials

The supporting information can be downloaded at: https://www.mdpi.com/article/10.3390/molecules27217538/s1. References [11,20,21,25,32,33] are cited in the supplementary materials.
