# Supplementary material for: Identification of Boronate-Containing Diarylpyrimidine Derivatives as Novel HIV-1 Non-Nucleoside Reverse Transcriptase Inhibitors
Source: Molecules. 2022 Nov 3;27(21):7538. doi: 10.3390/molecules27217538 (PMC9657321; doi:10.3390/molecules27217538)
Supplement: Supplementary file 1 [file molecules-27-07538-s001.zip › Supplementary Information-revise.pdf]

Identification of Boronate-Containing Diarylpyrimidine Derivatives as Novel HIV-1 Non-nucleoside Reverse Transcriptase Inhibitors

Da Feng [1,†], Hao Lin [1,†], Liyang Jiang [1], Zhao Wang [1], Zhongxia Zhou [1,3], Erik De Clercq [2], Christophe Pannecouque [2], Dongwei Kang [1,4,*], Peng Zhan [1,4,*] and Xinyong Liu [1,4,*]

[1] Department of Medicinal Chemistry, Key Laboratory of Chemical Biology (Ministry of Education), School of Pharmaceutical Sciences, Cheeloo College of Medicine, Shandong University, 44 West Culture Road, 250012 Jinan, China

[2] Rega Institute for Medical Research, Laboratory of Virology and Chemotherapy, K.U. Leuven, Herestraat 49 Postbus 1043 (09.A097), B-3000 Leuven, Belgium

[3] Department of Pharmacy, Shandong Cancer Hospital and Institute, Shandong First Medical University and Shandong Academy of Medical Sciences, Jinan 250117, China

[4] China-Belgium Collaborative Research Center for Innovative Antiviral Drugs of Shandong Province, 44 West Culture Road, 250012 Jinan, China

* Correspondence: kangdongwei@126.com (D.K.); zhanpeng1982@sdu.edu.cn (P.Z.); xinyongl@sdu.edu.cn (X.L.)

† These authors contributed equally to this work.

Supplemental Information

1. Chemistry

The synthetic protocols for the starting materials **1a**–**j** and **2a**–**c** are outlined in **Schemes S1** and **S2**, respectively. All derivatives were prepared by well-established methods as described in our previous articles. Commercially available 2,4-dichloropyrimidines **S1a**−**j** were selected as the starting materials, which were reacted with 4-hydroxy-3,5-dimethylbenzonitrile or 4-hydroxy-3,5-dimethylbenzaldehyde through nucleophilic substitution to afford **1a**−**j** and **S2a**−**c.** Then, **S2a**−**c** were treated with $(EtO)_2P(O)CH_2CN$ in the presence of *t*-BuOK to give **2a**−**c**.

Scheme S1. Synthesis of **1a–j**.[a]

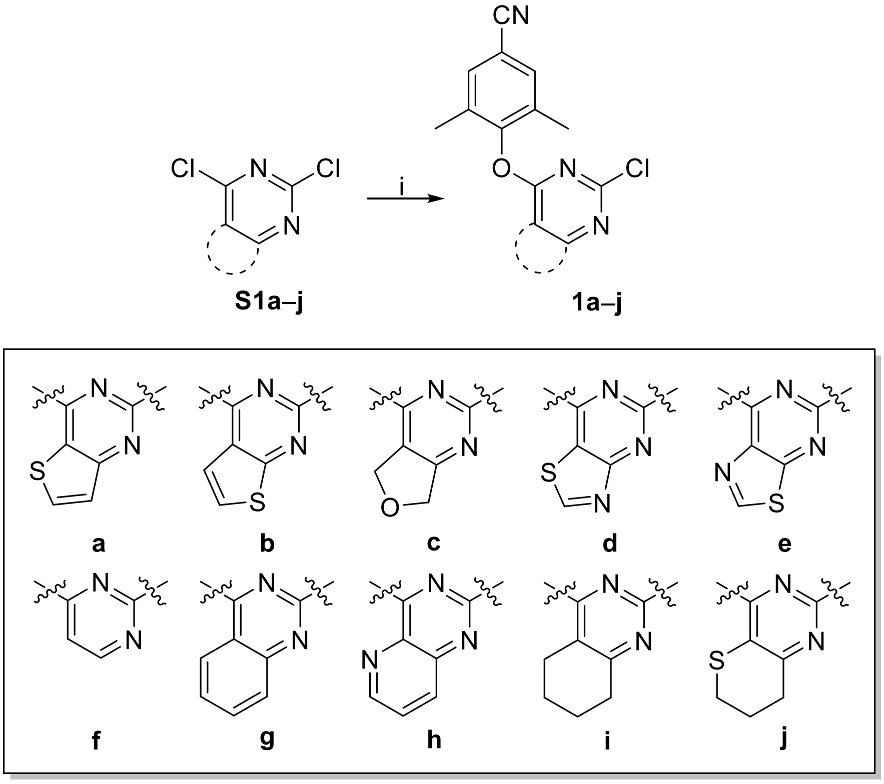

[a]Reagents and conditions: i) 3,5-dimethyl-4-hydroxybenzonitrile, K_2CO_3, DMF, r.t.

Scheme S2. Synthesis of **2a–c**.[a]

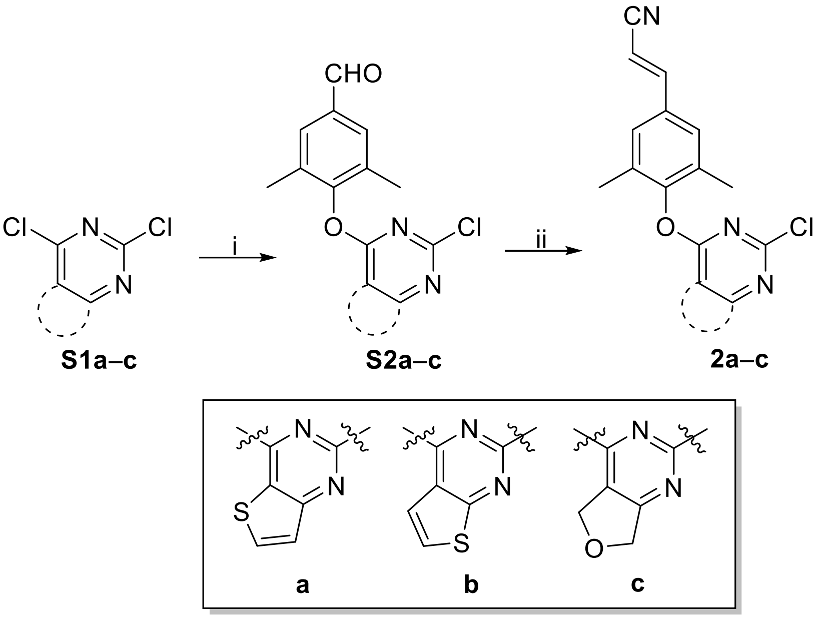

[a]Reagents and conditions: i) 4-hydroxy-3,5-dimethylbenzaldehyde, K_2CO_3, DMF, r.t.; ii) $(EtO)_2P(O)CH_2CN$, t-BuOK, THF, 0 °C to r.t.

2. Synthesis

All melting points were determined on a micro melting point apparatus (RY-1G, Tianjin Tian Guang Optical Instruments). ^{1}H NMR and ^{13}C NMR spectra were recorded in DMSO-d6 on a Bruker AV-400 spectrometer with tetramethylsilane (TMS) as the internal standard; signals are abbreviated as s (singlet), d (doublet), t (triplet), and m (multiplet). Chemical shifts are reported in δ values (ppm) from TMS, and coupling constants are given in hertz (Hz). The mass spectra were measured in AG1313A Standard LC Au-tosampler (Agilent). All reactions were routinely monitored by thin layer chromatog-raphy (TLC) on Silica Gel GF254 for TLC (Merck), and spots were visualized with iodine vapor or by irradiation with UV light (λ = 254 and 365 nm). Flash column chromatog-raphy was performed on columns packed with Silica Gel (Qingdao Haiyang Chemical Company). Solvents were purified and dried by standard methods. Concentration of the reaction solutions involved the use of rotary evaporator

at reduced pressure.

*2.1 General Procedure for the Compounds **1a-j**.*

4-((2-Chlorothieno[3,2-*d*]pyrimidin-4-yl)oxy)-3,5-dimethylbenzonitrile (1a) [1]

A reaction mixture of 4-hydroxy-3,5-dimethylbenzonitrile (0.15 g, 1 mmol) and potassium carbonate (0.17 g, 1.2 mmol) in 3 mL of DMF was stirred at 25 °C for 15 min, and then 2,4-dichlorothiopheno[3,2-*d*]pyrimidine (**S1a**, 0.21 g, 1 mmol) was added to it. Stirring was continued for an additional 2 h (monitored by TLC), then the mixture was poured into ice water and left to stand for 1 h. The precipitated white solid was collected by filtration, washed with cold water, and recrystallized in DMF−H_2O to provide the desired product **1a** as a white solid in 93% yield, mp 258−260 °C. ESI-MS: m/z 316.3 $[M + H]^+$. $C_{15}H_{10}ClN_3OS$ (315.02)

The synthetic procedures for target compounds **1b–j** were similar to that of **1a**.

4-((2-chlorothieno[2,3-*d*]pyrimidin-4-yl)oxy)-3,5-dimethylbenzonitrile (1b) [2] White solid in 91% yield. mp 269-271°C. ESI-MS: m/z 316.0 $[M + H]^+$. $C_{15}H_{10}ClN_3OS$ (315.02)

4-((2-Chloro-5,7-dihydrofuro[3,4-*d*]pyrimidin-4-yl)oxy)-3,5-dimethylbenzonitrile (1c) [3] White solid in 88% yield. mp 180−183 °C. ^1H NMR (400 MHz, DMSO-d_6) δ 7.69 (s, 2H), 4.97 (s, 2H), 4.80 (s, 2H), 2.10 (s, 6H). ESI-MS: m/z 302.07 $[M + H]^+$. $C_{15}H_{12}ClN_3O_2$ (301.06)

4-((5-chlorothiazolo[4,5-*d*]pyrimidin-7-yl)oxy)-3,5-dimethylbenzonitrile (1d) White solid in 94% yield. mp 250-253°C. ^1H NMR (400 MHz, DMSO-d_6) δ 9.60 (s, 1H), 7.78 (s, 2H), 2.14 (s, 6H). ESI-MS: m/z 317.03 $[M + H]^+$. $C_{14}H_9ClN_4OS$ (316.02)

4-((5-chlorothiazolo[5,4-*d*]pyrimidin-7-yl)oxy)-3,5-dimethylbenzonitrile (1e) White solid in 93% yield. mp 265-268°C. ^{1}H NMR (400 MHz, DMSO-d_6) δ 9.06 (s, 1H), 7.67 (s, 2H), 2.14 (s, 6H). ESI-MS: m/z 317.03 [M + H]$^{+}$. $C_{14}H_9ClN_4OS$ (316.02).

4-((2-chloropyrimidin-4-yl)oxy)-3,5-dimethylbenzonitrile (1f) [4] White solid in 90% yield. mp 188-190°C. ^{1}H NMR (400 MHz, DMSO-d_6): δ 8.70 (d, *J* = 5.7 Hz, 1H), 7.75 (s, 2H), 7.32 (d, *J* = 5.7 Hz, 1H), 2.10 (s, 6H). ESI-MS m/z: 260.3 [M + H]$^{+}$. $C_{13}H_{10}ClN_3O$ (259.05)

4-((2-chloroquinazolin-4-yl)oxy)-3,5-dimethylbenzonitrile (1g) White solid in 90% yield. mp 245-247°C. ^{1}H NMR (400 MHz, DMSO-d_6) δ 8.46 (d, *J* = 8.2 Hz, 1H), 8.12 (t, *J* = 7.9 Hz, 1H), 7.98 (d, *J* = 8.5 Hz, 1H), 7.85 (t, *J* = 7.7 Hz, 1H), 7.75 (s, 2H), 2.15 (s, 6H).

4-((2-chloropyrido[3,2-d]pyrimidin-4-yl)oxy)-3,5-dimethylbenzonitrile (1h) [4] White solid in 79% yield. mp 126-128°C. ^{1}H NMR (400 MHz, DMSO-*d_6*) δ 8.59 (d, *J* = 4.3 Hz, 1H), 7.78 – 7.77 (m, 1H), 7.75 (s, 2H), 7.73 – 7.71 (m, 1H), 2.12 (s, 6H). ESI-MS: m/z 311.6 [M + H]$^{+}$. $C_{16}H_{11}ClN_4O$ (310.06)

4-((2-chloro-5,6,7,8-tetrahydroquinazolin-4-yl)oxy)-3,5-dimethylbenzonitrile (1i) [3] White solid in 88% yield. mp 175−177 °C. ^{1}H NMR (400 MHz, DMSO-d_6) δ 7.65 (s, 2H), 2.56 (d, *J* = 16.0 Hz, 4H), 2.06 (s, 6H), 1.72 (t, *J* = 3.3 Hz, 4H). ESI-MS: m/z 314.11 [M + H]$^{+}$. $C_{17}H_{16}ClN_3O$ (313.10)

4-((2-chloro-7,8-dihydro-6H-thiopyrano[3,2-*d*]pyrimidin-4-yl)oxy)-3,5-dimethylbenzonitrile (1j) [5] White solid in 66% yield. mp 254−255 °C. ^{1}H NMR (400 MHz, DMSO-d_6) δ: 7.73 (s, 2H), 3.16 (d, *J* = 5.6 Hz, 2H), 2.92 (t, *J* = 6.2 Hz, 2H),

2.23 − 2.13 (m, 2H), 2.07 (s, 6H). ESI-MS: m/z 332.4 $[M + H]^+$. $C_{16}H_{14}ClN_3OS$ (331.1)

*2.2 General Procedure for the Compounds **2a-c**.*

4-((2-chlorothieno[3,2-*d*]pyrimidin-4-yl)oxy)-3,5-dimethylbenzaldehyde (S2a) [2] A mixture of 4-hydroxy-3,5-dimethylbenzaldehyde (0.88g, 5.85 mmol) and potassium carbonate (1.35 g, 9.75 mmol) in 15 mL of DMF was stirred at 25 °C for 15 min, and then 2,4-dichlorothiopheno[3,2-*d*]pyrimidine (**S1a**, 1.0 g, 4.88 mmol) was added to it. The mixture was stirred for another 1.5 h (monitored by TLC), then poured into ice−water (30 mL) and left to stand for 1 h. The precipitated white solid was collected by filtration, washed with cold water, and recrystallized from DMF−H_2O to provide **S2a** as a white solid in 90% yield, mp 285−287 °C. ESI-MS m/z 319.03 $[M + H]^+$. $C_{15}H_{11}ClN_2O_2S$ (318.02)

(*E*)-3-(4-((2-chlorothieno[3,2-*d*]pyrimidin-4-yl)oxy)-3,5-dimethylphenyl)acrylonitrile (2a) [2] A mixture of $(EtO)_2P(O)CH_2CN$ (0.67 g, 3.76 mmol) and t-BuOK (0.71 g, 6.27 mmol) in THF (10 mL) was stirred for 1 h at 0 °C, and then a solution of **S2a** (1.0 g, 3.14 mmol) in THF (10 mL) was slowly added to it over 1 h (monitored by TLC). The mixture was poured into ice−water (20 mL), and the precipitate was collected, washed with water, and dried to afford **2a** as a white solid in 86% yield. ^1H NMR (400 MHz, DMSO-d_6): δ 8.17 (d, 1H, J = 5.4 Hz), 7.60 (d, 1H, J = 16.7 Hz), 7.49 (s, 2H), 7.45 (d, 2H, J = 8.1 Hz), 7.32 (s, 2H), 7.25 (s, 1H), 6.45 (d, 1H, J = 16.7 Hz), 2.09 (s, 6H). ESI-MS m/z 342.05 $[M + H]^+$. $C_{17}H_{12}ClN_3OS$ (341.04)

4-((2-Chlorothieno[2,3-*d*]pyrimidin-4-yl)oxy)-3,5-dimethylbenzaldehyde (S2b) [6] White solid in 85% yield, mp 263−265 °C. ESI−MS: m/z 319.4 $[M + H]^+$.

$C_{15}H_{11}ClN_2O_2S$ (318.02)

(*E*)-3-(4-((2-Chlorothieno[2,3-*d*]pyrimidin-4-yl)oxy)-3,5-dimethylphenyl)acrylonitrile (2b) [7] White solid in 72% yield, mp 235−237 °C. ESI−MS: m/z 342.4 $[M + H]^+$. $C_{17}H_{12}ClN_3OS$ (341.04)

4-((2-chloro-5,7-dihydrofuro[3,4-*d*]pyrimidin-4-yl)oxy)-3,5-dimethylbenzaldehyde (S2c) [8] White solid in 92% yield, mp 241−243 °C. ^1H NMR (400 MHz, DMSO-d_6): δ 9.83 (s, 1H), 7.44 (s, 2H), 4.92 (s, 2H), 4.76 (s, 2H), 2.06 (s, 6H). ESI-MS m/z: 305.2 $[M + H]^+$. $C_{15}H_{13}ClN_2O_3$ (304.06)

(*E*)-3-(4-((2-Chloro-5,7-dihydrofuro[3,4-*d*]pyrimidin-4-yl)oxy)-3,5-dimethylphenyl)acrylonitrile (2c) [9] White solid in 74% yield, mp 256−258 °C. ^1H NMR (400 MHz, DMSO-d_6): δ 7.69 (d, J = 16.7 Hz, 1H), 7.44 (s, 2H), 6.50 (d, J = 16.4 Hz, 1H), 4.92 (s, 2H), 4.76 (s, 2H), 2.06 (s, 6H). ESI-MS m/z: 328.3 $[M + H]^+$. $C_{17}H_{14}ClN_3O_2$ (327.08)

3. ^1H-NMR and ^{13}C-NMR Spectra for Representative Target Compounds

3.1. ^1H-NMR and ^{13}C-NMR Spectra for 3a

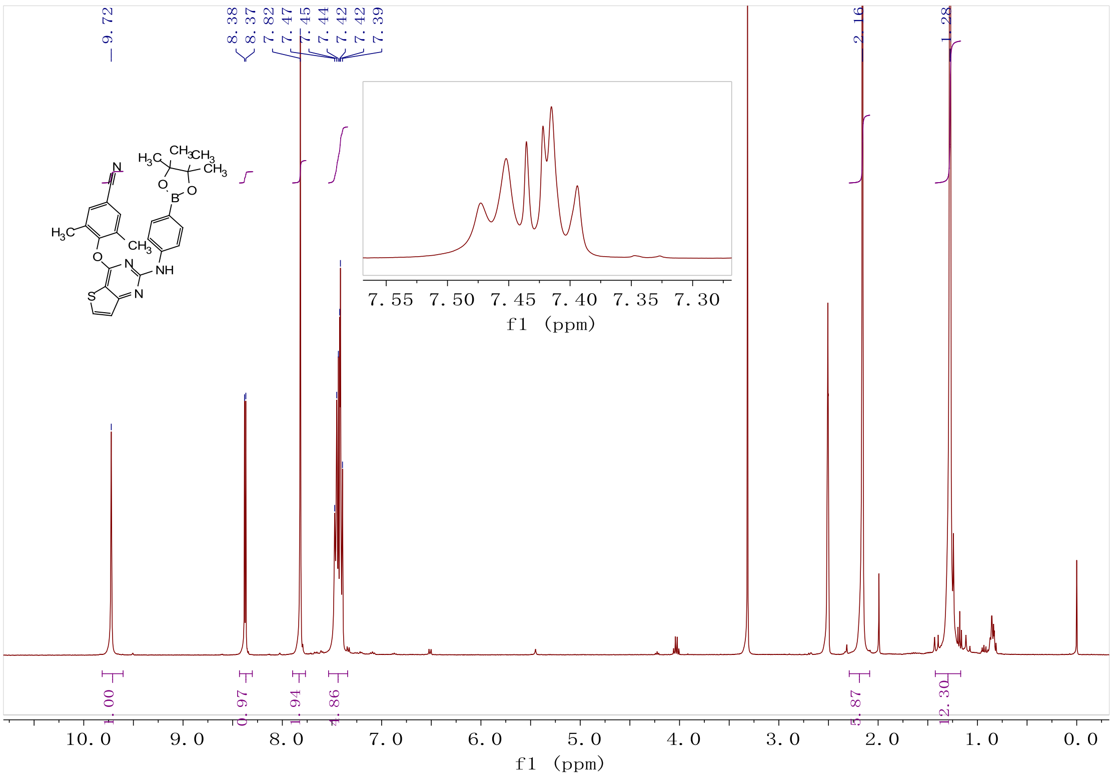

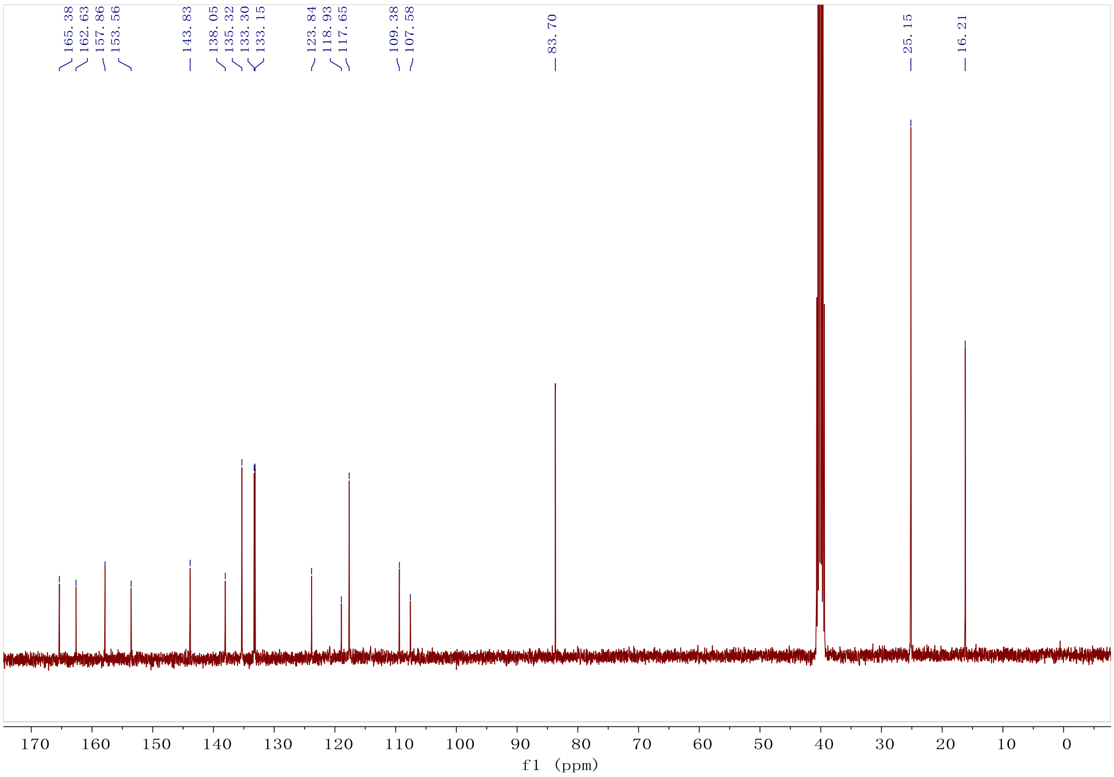

3.2. ^{1}H-NMR and ^{13}C-NMR Spectra for 3c

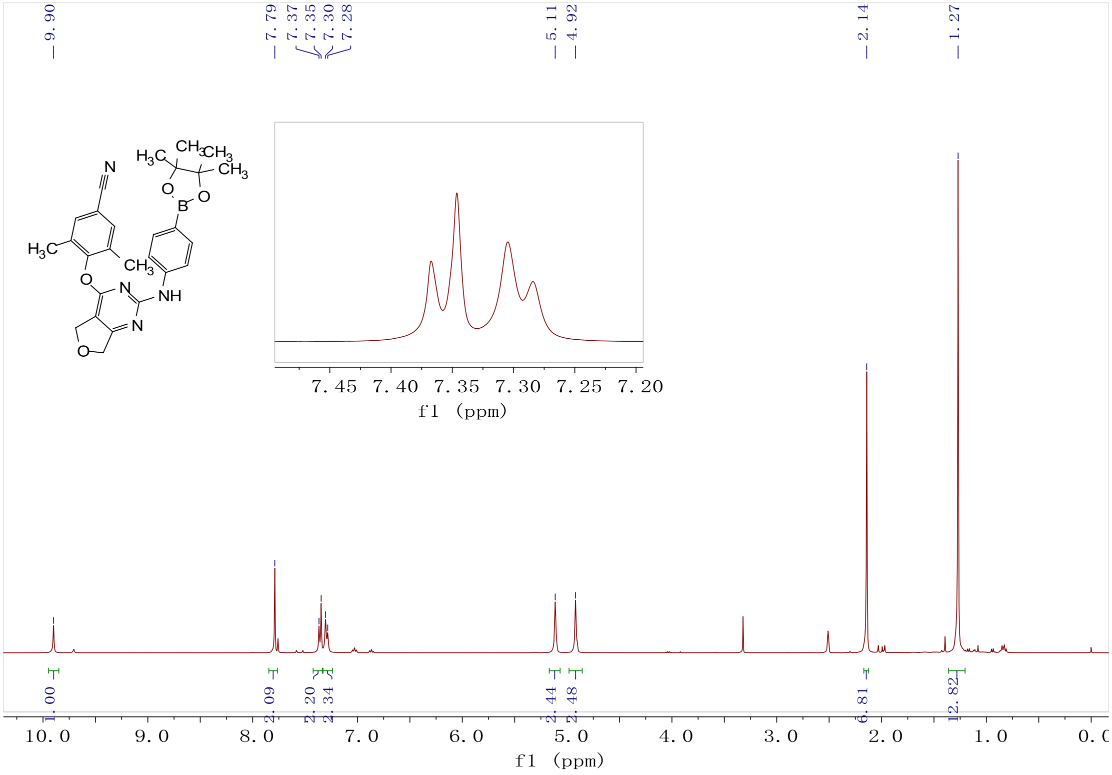
9.90
7.79
7.37
7.35
7.30
7.28
5.11
4.92
2.14
1.27
N
H_3C
CH_3
O
N
N
NH
O
B
O
H_3C
CH_3
CH_3
CH_3
7.45 7.40 7.35 7.30 7.25 7.20
f1 (ppm)
1.00
2.09
2.20
2.34
2.44
2.48
6.81
12.82
10.0 9.0 8.0 7.0 6.0 5.0 4.0 3.0 2.0 1.0 0.0
f1 (ppm)

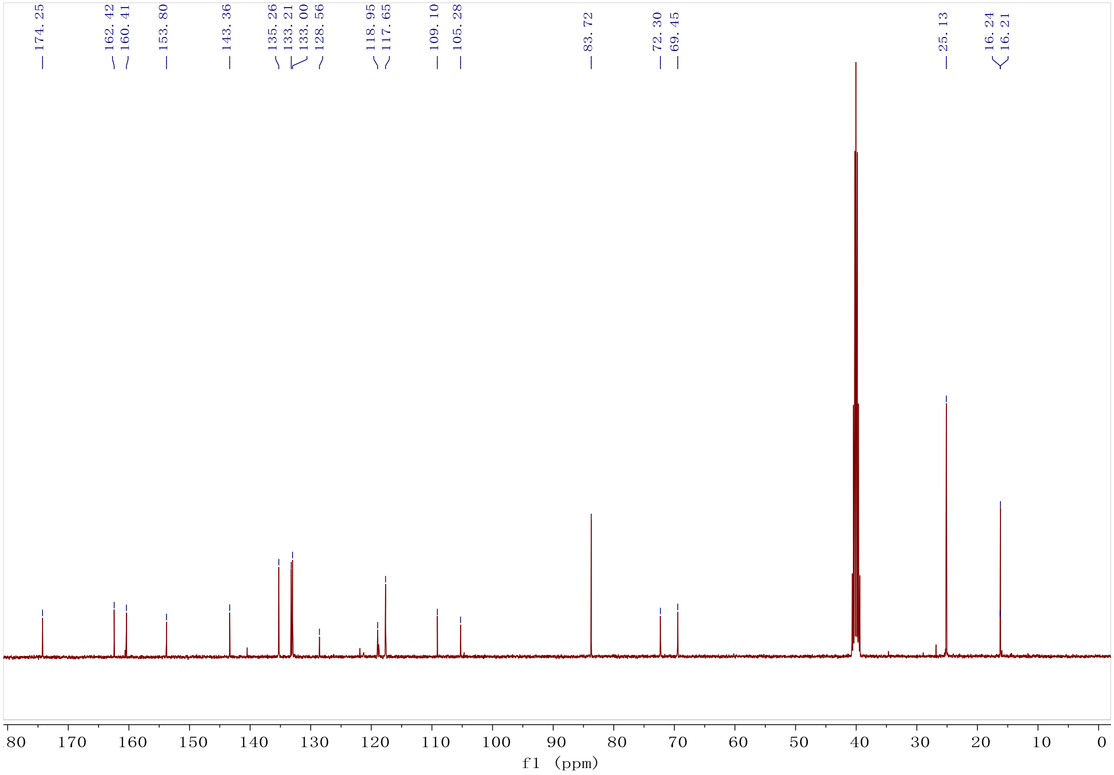
174.25
162.42
160.41
153.80
143.36
135.26
133.21
133.00
128.56
118.95
117.65
109.10
105.28
83.72
72.30
69.45
25.13
16.24
16.21
180 170 160 150 140 130 120 110 100 90 80 70 60 50 40 30 20 10 0
f1 (ppm)

3.3. ^{1}H-NMR and ^{13}C-NMR Spectra for 3f

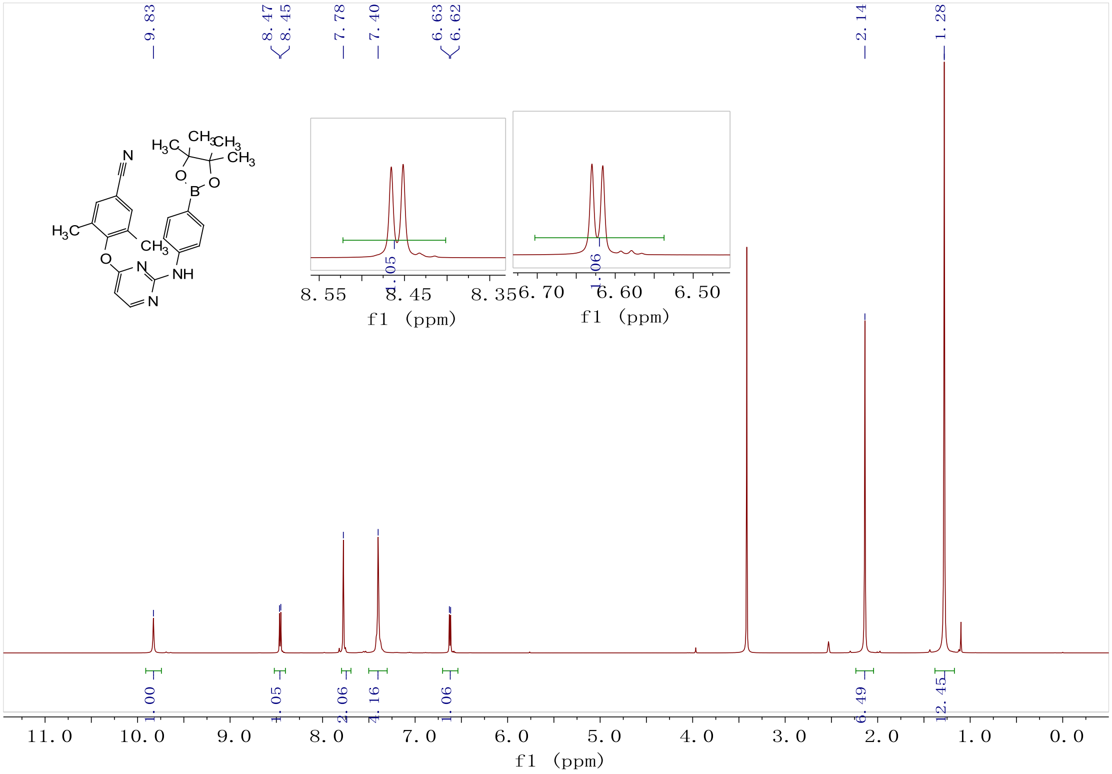

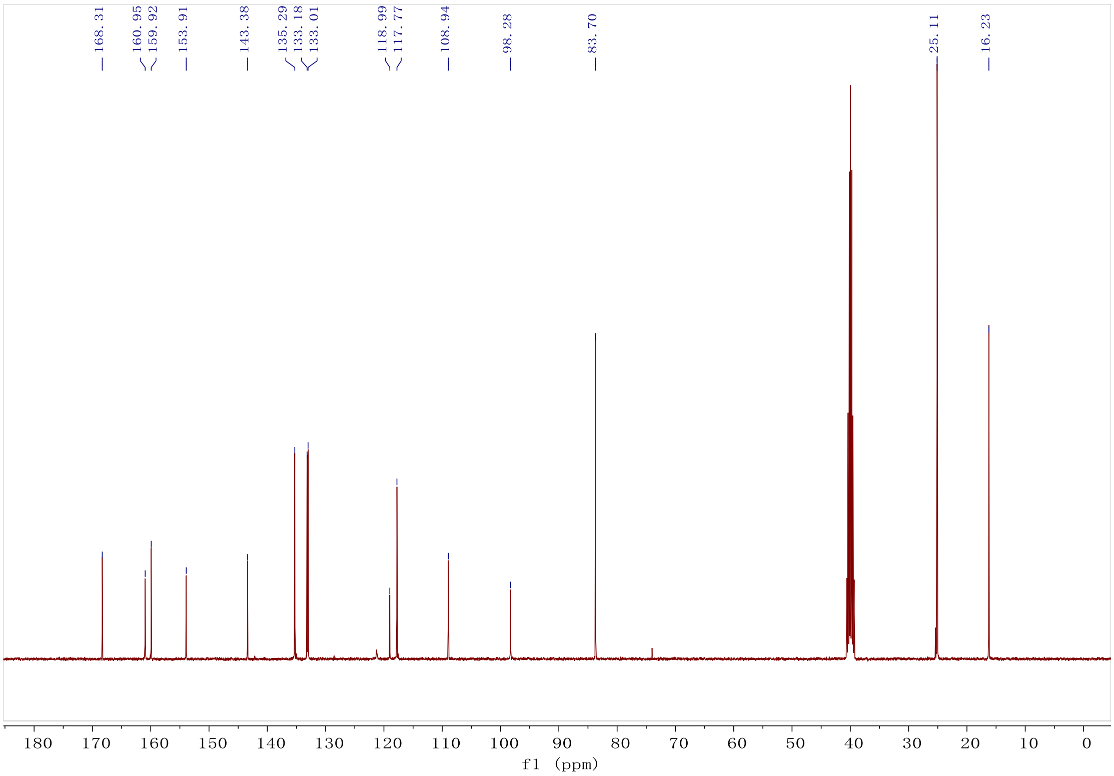

3.4. ^{1}H-NMR and ^{13}C-NMR Spectra for 3h

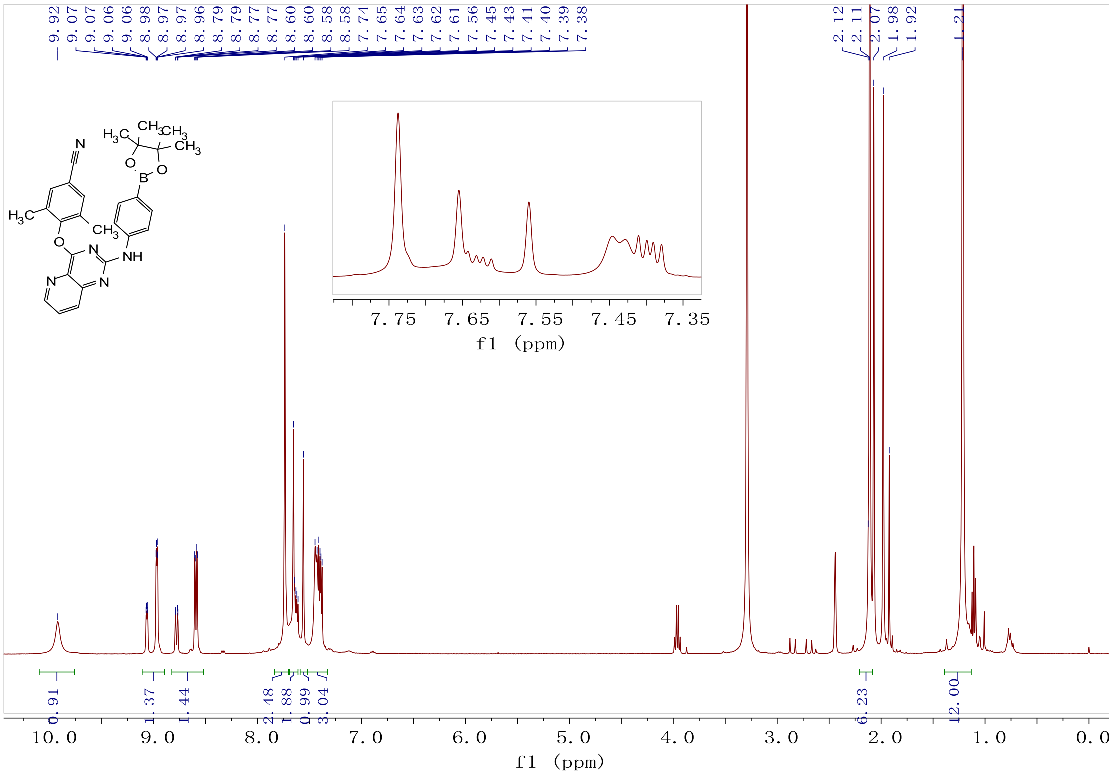
9.92
9.07
9.07
9.06
9.06
8.98
8.97
8.97
8.96
8.79
8.79
8.77
8.77
8.60
8.60
8.58
8.58
7.74
7.65
7.64
7.63
7.62
7.61
7.56
7.45
7.43
7.41
7.40
7.39
7.38
2.12
2.11
2.07
1.98
1.92
1.21
N
O
B
O
NH
N
N
N
O
7.75
7.65
7.55
7.45
7.35
f1 (ppm)
0.91
1.37
1.44
2.48
1.88
0.99
3.04
6.23
12.00
10.0
9.0
8.0
7.0
6.0
5.0
4.0
3.0
2.0
1.0
0.0
f1 (ppm)

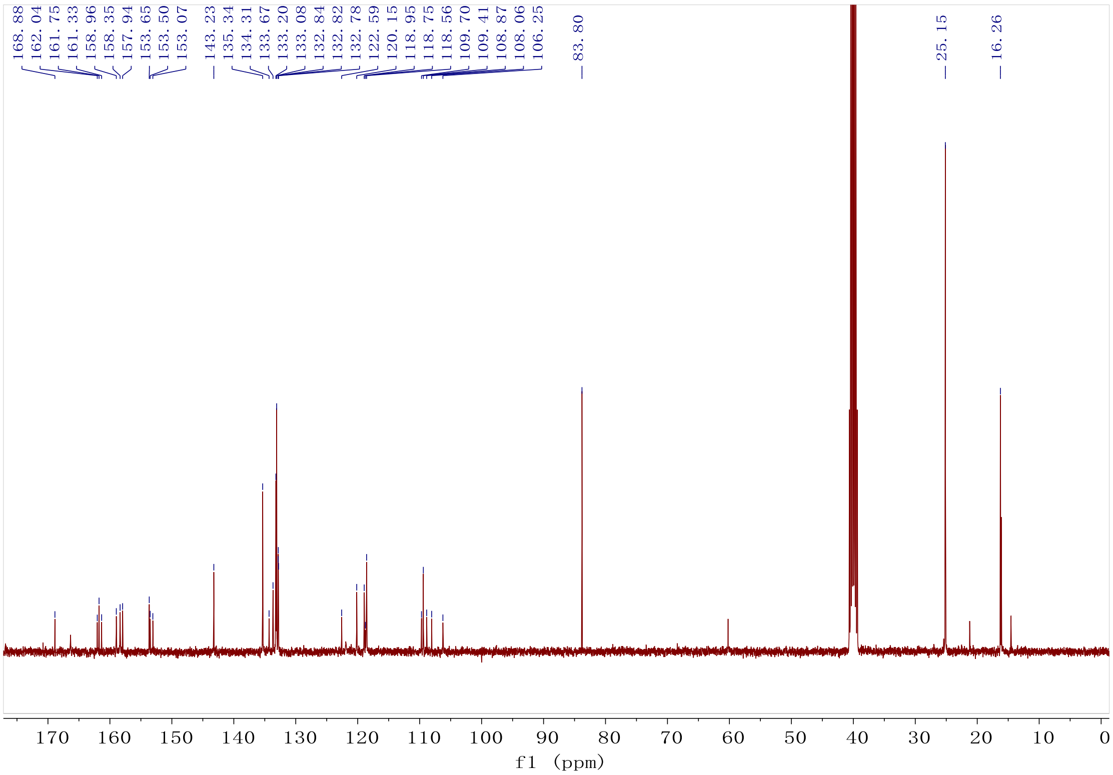
168.88
162.04
161.75
161.33
158.96
158.35
157.94
153.65
153.50
153.07
143.23
135.34
134.31
133.67
133.20
133.08
132.84
132.82
132.78
122.59
120.15
118.95
118.75
118.56
109.70
109.41
108.87
108.06
106.25
83.80
25.15
16.26
170
160
150
140
130
120
110
100
90
80
70
60
50
40
30
20
10
0
f1 (ppm)

3.5. ^{1}H-NMR and ^{13}C-NMR Spectra for 4a

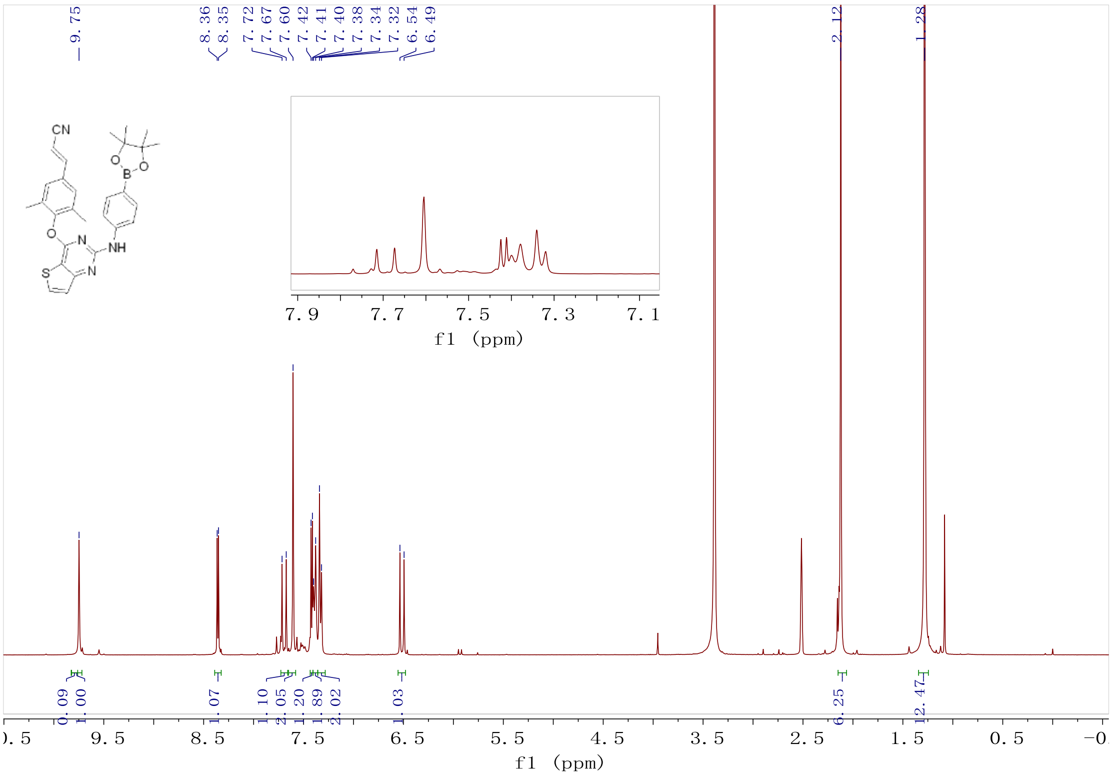

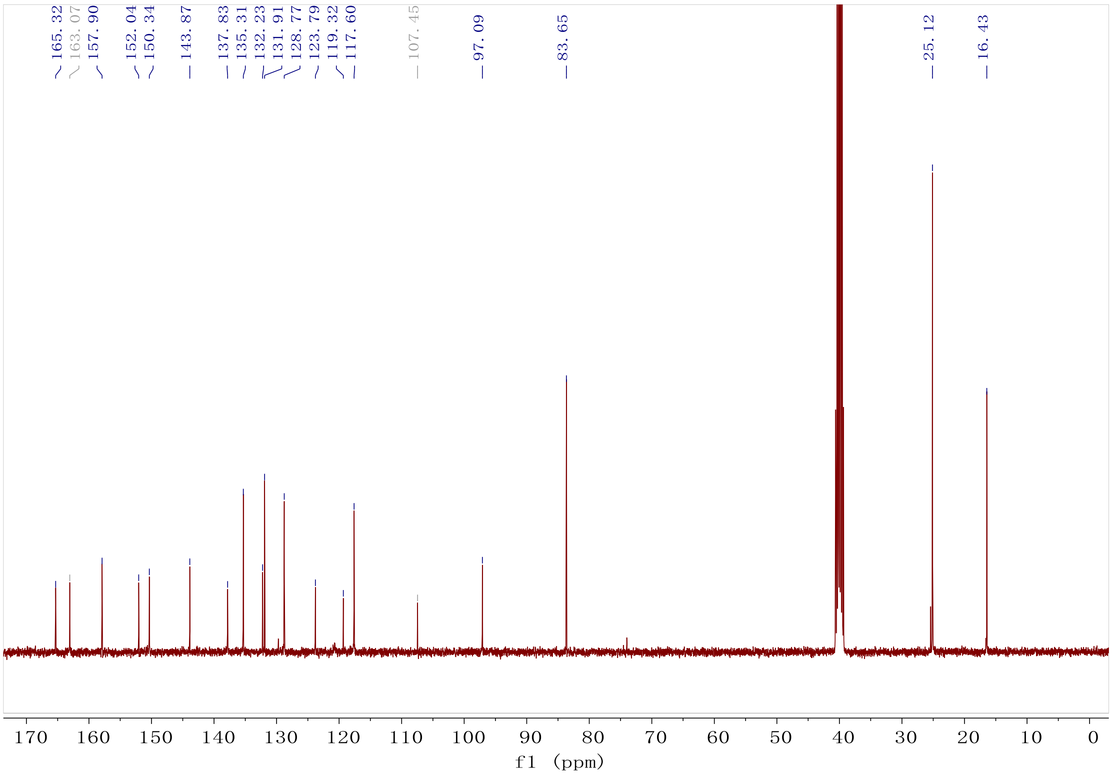

3.6. ^{1}H-NMR and ^{13}C-NMR Spectra for 4b

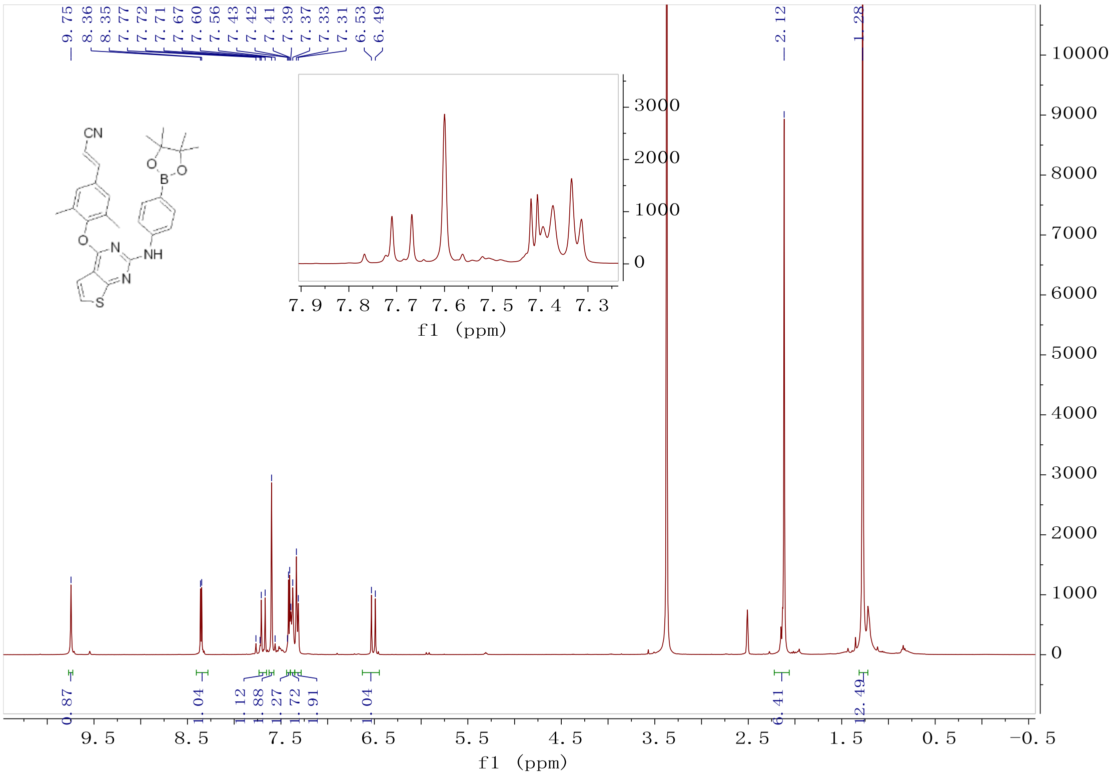

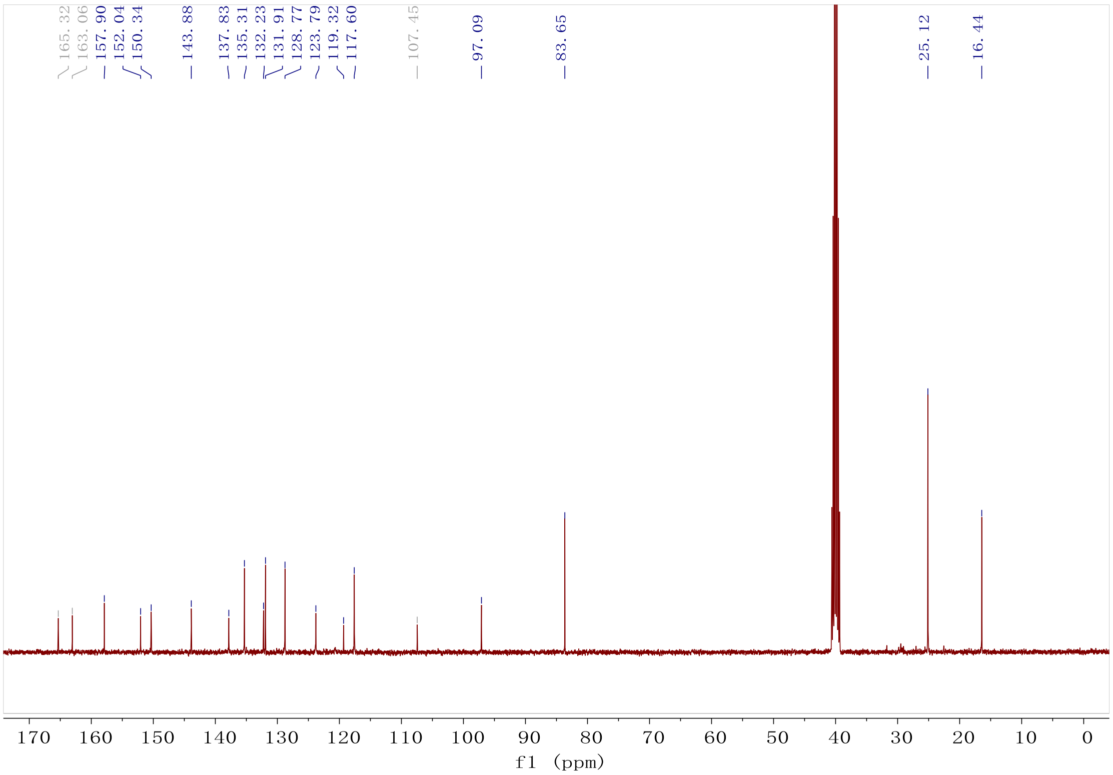

3.7. ^{1}H-NMR Spectra for 4c

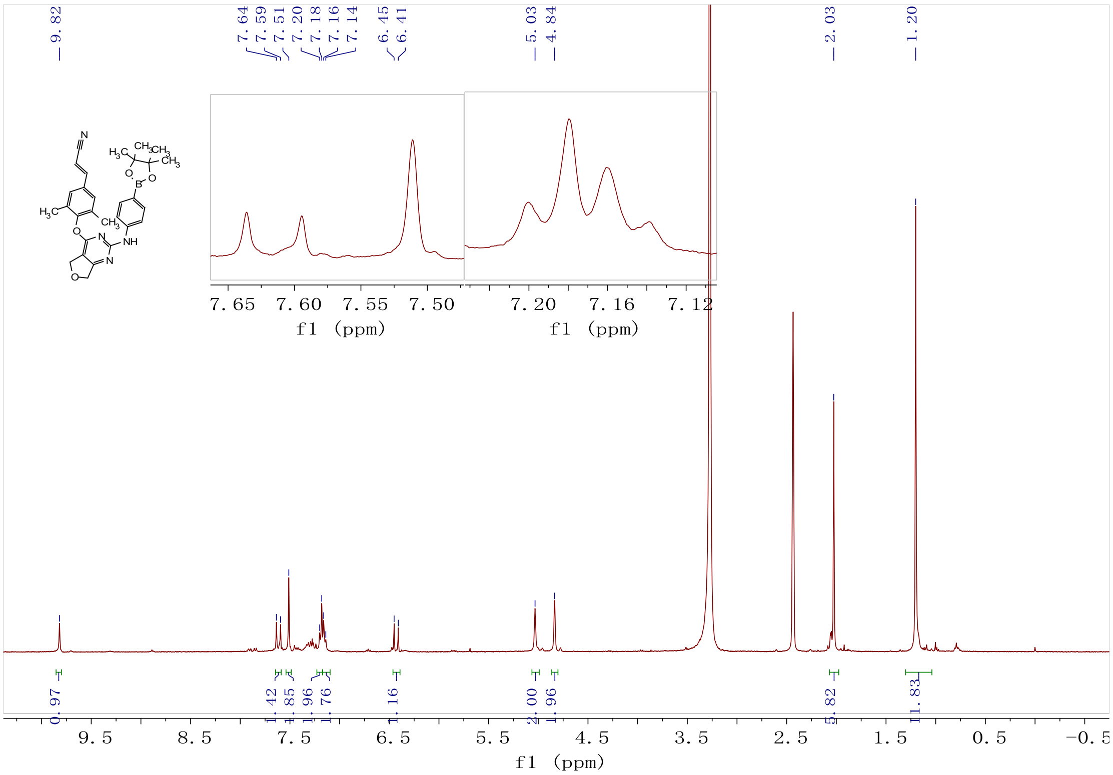

References

1. Kang, D.; Fang, Z.; Li, Z.; Huang, B.; Zhang, H.; Lu, X.; Xu, H.; Zhou, Z.; Ding, X.; Daelemans, D.; De Clercq, E.; Pannecouque, C.; Zhan, P.; Liu, X., Design, Synthesis, and Evaluation of Thiophene[3,2-d]pyrimidine Derivatives as HIV-1 Non-nucleoside Reverse Transcriptase Inhibitors with Significantly Improved Drug Resistance Profiles. *Journal of medicinal chemistry* **2016,** 59, (17), 7991-8007.
2. Kang, D.; Fang, Z.; Huang, B.; Lu, X.; Zhang, H.; Xu, H.; Huo, Z.; Zhou, Z.; Yu, Z.; Meng, Q.; Wu, G.; Ding, X.; Tian, Y.; Daelemans, D.; De Clercq, E.; Pannecouque, C.; Zhan, P.; Liu, X., Structure-Based Optimization of Thiophene[3,2-d]pyrimidine Derivatives as Potent HIV-1 Non-nucleoside Reverse Transcriptase Inhibitors with Improved Potency against Resistance-Associated Variants. *Journal of medicinal chemistry* **2017,** 60, (10), 4424-4443.
3. Kang, D.; Zhang, H.; Wang, Z.; Zhao, T.; Ginex, T.; Luque, F. J.; Yang, Y.; Wu, G.; Feng, D.; Wei, F.; Zhang, J.; De Clercq, E.; Pannecouque, C.; Chen, C. H.; Lee, K. H.; Murugan, N. A.; Steitz, T. A.; Zhan, P.; Liu, X., Identification of Dihydrofuro[3,4- d]pyrimidine Derivatives as Novel HIV-1 Non-Nucleoside Reverse Transcriptase Inhibitors with Promising Antiviral Activities and Desirable Physicochemical Properties. *Journal of medicinal chemistry* **2019,** 62, (3), 1484-1501.
4. Kang, D.; Ruiz, F. X.; Sun, Y.; Feng, D.; Jing, L.; Wang, Z.; Zhang, T.; Gao, S.; Sun, L.; De Clercq, E.; Pannecouque, C.; Arnold, E.; Zhan, P.; Liu, X., 2,4,5-Trisubstituted Pyrimidines as Potent HIV-1 NNRTIs: Rational Design, Synthesis, Activity Evaluation, and Crystallographic Studies. *Journal of medicinal chemistry* **2021,** 64, (7), 4239-4256.
5. Kang, D.; Feng, D.; Sun, Y.; Fang, Z.; Wei, F.; De Clercq, E.; Pannecouque, C.; Liu, X.; Zhan, P., Structure-Based Bioisosterism Yields HIV-1 NNRTIs with Improved Drug-Resistance Profiles and Favorable Pharmacokinetic Properties. *Journal of medicinal chemistry* **2020,**

63, (9), 4837-4848.
6. Kang, D.; Sun, Y.; Feng, D.; Gao, S.; Wang, Z.; Jing, L.; Zhang, T.; Jiang, X.; Lin, H.; De Clercq, E.; Pannecouque, C.; Zhan, P.; Liu, X., Development of Novel Dihydrofuro[3,4-d]pyrimidine Derivatives as HIV-1 NNRTIs to Overcome the Highly Resistant Mutant Strains F227L/V106A and K103N/Y181C. *Journal of medicinal chemistry* **2022,** 65, (3), 2458-2470.
